# The RNA-binding protein Rbm38 is dispensable during pressure overload-induced cardiac remodeling in mice

**DOI:** 10.1371/journal.pone.0184093

**Published:** 2017-08-29

**Authors:** Maarten M. G. van den Hoogenhof, Ingeborg van der Made, Abdelaziz Beqqali, Nina E. de Groot, Amin Damanafshan, Ralph J. van Oort, Yigal M. Pinto, Esther E. Creemers

**Affiliations:** Department of Experimental Cardiology, Academic Medical Center, Amsterdam, The Netherlands; University of California Davis, UNITED STATES

## Abstract

The importance of tightly controlled alternative pre-mRNA splicing in the heart is emerging. The RNA binding protein Rbm24 has recently been identified as a pivotal cardiac splice factor, which governs sarcomerogenesis in the heart by controlling the expression of alternative protein isoforms. Rbm38, a homolog of Rbm24, has also been implicated in RNA processes such as RNA splicing, RNA stability and RNA translation, but its function in the heart is currently unknown. Here, we investigated the role of Rbm38 in the healthy and diseased adult mouse heart. In contrast to the heart- and skeletal muscle-enriched protein Rbm24, Rbm38 appears to be more broadly expressed. We generated somatic Rbm38 -/- mice and show that global loss of Rbm38 results in hematopoietic defects. Specifically, Rbm38 -/- mice were anemic and displayed enlarged spleens with extramedullary hematopoiesis, as has been shown earlier. The hearts of Rbm38 -/- mice were mildly hypertrophic, but cardiac function was not affected. Furthermore, Rbm38 deficiency did not affect cardiac remodeling (i.e. hypertrophy, LV dilation and fibrosis) or performance (i.e. fractional shortening) after pressure-overload induced by transverse aorta constriction. To further investigate molecular consequences of Rbm38 deficiency, we examined previously identified RNA stability, splicing, and translational targets of Rbm38. We found that stability targets p21 and HuR, splicing targets Mef2d and Fgfr2, and translation target p53 were not altered, suggesting that these Rbm38 targets are tissue-specific or that Rbm38 deficiency may be counteracted by a redundancy mechanism. In this regard, we found a trend towards increased Rbm24 protein expression in Rbm38 -/- hearts. Overall, we conclude that Rbm38 is critical in hematopoiesis, but does not play a critical role in the healthy and diseased heart.

## Introduction

Heart disease is one of the major causes of death in the Western world, but the underlying molecular mechanisms are not entirely understood. Recently, it has become clear that RNA splicing underlies functional properties of the heart [1, 2]. Mutations in the RNA-binding protein (RBP) and splicing factor RNA-binding motif protein 20 (RBM20) cause an aggressive form of dilated cardiomyopathy (DCM) [3–5]. In addition, RNA-binding motif 24 (Rbm24) has recently been shown to play an important role in cardiac development and sarcomerogenesis through alternative splicing regulation of at least 68 genes [6]. Rbm38 (or RNPC1) is a homolog of Rbm24, but its function in the heart is currently unknown. Rbm38 shares several targets with Rbm24; for instance they both regulate the stability of p21 and p63 mRNA [7–11]. Furthermore, the *C Elegans* ortholog of Rbm24 and Rbm38, SUP-12, has been implicated in muscle-specific splicing of ADF/cofilin and fibroblast growth factor receptor 2 (Fgfr2) [12, 13]. Moreover, Rbm38 has been shown to regulate splicing of Fgfr2 in epithelial cells [14]. However, while Rbm24 is expressed specifically in heart- and skeletal muscle, Rbm38 appears to be expressed in a wider range of tissues and cell-types, but some discussion exists about its exact expression pattern. For instance, Miyamoto et al. show enriched expression of Rbm38 in heart- and skeletal muscle, while others have shown specificity of Rbm38 in erythrocytes [15, 16]. Rbm38 has been implicated, like many other RBPs, in the regulation of RNA processes such as RNA splicing, RNA stability, RNA translation, and microRNA accessibility to mRNA targets [10, 16–18]. In erythrocytes, Rbm38 has recently been identified as a critical regulator of cell maturation, where it mediates alternative splicing of regulators of blood cell maturation such as EPB41, Mef2d, and CYB5A [15, 18, 19]. Rbm38 knockout mice are, not surprisingly, susceptible to hematopoietic defects, as well as accelerated aging and spontaneous tumors [20].

Rbm38 is intimately connected with the p53 pathway, and is required for proper functioning of p53 [11, 21–24]. Along these lines, Rbm38 knockout mice have increased expression of p53 in multiple tissues and cells (i.e. spleen, thymus and embryonic fibroblasts), which likely contributes to their increased tumor penetrance [20]. In multiple types of cancer, e.g. breast- and colorectal cancer, Rbm38 expression is regulated, which indicates that Rbm38 function may represent a clinically relevant target [25, 26]. Several members of the p53 transcription factor family are targeted by Rbm38, either through RNA stability or RNA translation. Among these family members, Rbm38 stabilizes mRNAs of p21 and p73, while it destabilizes p63 mRNA [9, 11, 22]. Interestingly, phosphorylation of Rbm38 by glycogen synthase kinase 3 (GSK3) converts Rbm38 from a translational repressor to an activator [24]. Phosphorylated Rbm38 interacts with eukaryotic translation factor eIF4E, which facilitates translation of the p53 mRNA [24]. In addition, Rbm38 regulates several targets downstream of the p53-pathway, such as p21, DDIT4, LATS2, and Rbm38 itself, by inhibiting miRNA accessibility of their mRNAs [21]. Whether Rbm38 also regulates p53 levels in the heart is currently unknown, but potentially highly relevant since p53 was recently shown to exhibit anti-angiogenic properties in the heart. In this regard, blocking p53 expression in mice was shown to counteract maladaptive cardiac remodeling by stimulating Hif-1 mediated angiogenesis after sustained pressure overload [27].

Recent insight into the importance of alternative splicing in the heart has spurred the interest in uncovering the roles of RNA-binding proteins in the heart, but the role of Rbm38 has not yet been studied. This led us to hypothesize that Rbm38 is required for cardiac homeostasis and function. In this study we found that the expression of Rbm38 is dispensable in the heart, both at baseline and during pressure overload-induced cardiac remodeling. Furthermore, loss of Rbm38 might be compensated by upregulation of the closely related RBP Rbm24.

## Materials and methods

### Rbm38 knockout mouse generation

C57Bl/6N embryonic stem (ES) cells with a conditional Rbm38 knockout allele were obtained from the International Knockout Mouse Consortium (Knockout-first alleles, UC Davis, www.komp.org, MGI:1889294) and generated by the Wellcome Trust Sanger Institute [28]. The knockout allele consists of a neomycin and LacZ cassette, together with LoxP and Flp sites, flanking exons 3 and 4. Targeted ES cells were pronuclear injected in blastocysts (FVB background). After germline transmission, the neomycin and LacZ cassete were excised, by crossing with Flp-mice (FVB background). To get a total body (heterozygous) knockout, we crossed conditional Rbm38 +/- with CMV-Cre mice (FVB background). Rbm38 +/- were crossed with wildtype FVB mice at least 6 additional times to obtain a pure background. All animal studies were approved by the Institutional Animal Care and Use Committee of the University of Amsterdam (approval# DCA57AC-1), and in accordance with the guidelines of this institution and the Directive 2010/63/EU of the European Parliament.

### Echocardiography

Echocardiography was performed as previously described [29]. In short, anesthetized mice (2.5% isoflurane) were subjected to transthoracic two-dimensional echocardiography (Vevo 770 Ultrasound, Visual Sonics), using a 30-Mhz linear array transducer to measure LV function and dimensions. Left ventricular internal diameters were measured using M-mode tracings in parasternal short axis view at end-systole and end-diastole.

### Transverse aorta constriction (TAC) surgery

TAC surgery was performed to induce sustained LV pressure overload, as previously described [29]. 8–10 weeks old wildtype and Rbm38 knockout mice (FVB) were subjected to TAC or sham surgeries. In short, the aortic arch was constricted with a ligature between the truncus brachiocephalus and the arteria carotis communis sinistra around a 27G needle, which was removed immediately after constriction to (partially) restore blood flow. Sham-operated animals underwent the exact same procedure, but without the aortic ligation. As analgesia, mice were injected subcutaneously with Carprofen (Pfizer, 5 mg/kg) and Temgesic (0.05 mg/kg), prior to surgery and the first 3 days after surgery. While under anesthesia from echocardiography, all mice were sacrificed by cervical dislocation 7 weeks after surgery. Number of animals per group: wildtype sham n = 7, wildtype TAC n = 8, Rbm38 -/- sham n = 3, Rbm38 -/- TAC n = 7.

### Primary cell isolation

Neonatal rat cardiomyocytes and fibroblasts were isolated as previously described [30]. Briefly, hearts of 1–3 day old Wistar hearts were excised, ventricles were cut into small pieces, and left overnight to rotate in Hank’s Balanced Salt Solution (HBSS)(Gibco) with 1 mg/ml Trypsin (Affymetrix) at 4°C. Cells were dissociated the next day with 1 mg/ml collagenase type 2 (Worthington) in HBSS. Total cell suspension was pre-plated to separate cardiomyocytes from fibroblasts. After 2 hours, non-adherent cells (cardiomyocytes) were collected, counted, and plated on fibronectin (Corning) coated plates (5 x 10^6 cells/10 cm plate). Adherent cells (fibroblasts) were passaged at least once to obtain a pure fibroblast population. Adult mouse cardiomyocytes were isolated by enzymatic dissociation on a Langendorff-setup as previously described [31], and plated on laminin (Sigma) coated coverslips.

### RNA isolation, cDNA preparation and (q)RT-PCR

RNA was isolated using TRIreagent (Sigma-Aldrich) according to manufacturer’s protocol. Human RNA tissue panel was acquired from Clontech (Human Total RNA Master Panel II). cDNA was synthesized using SuperScript II (Invitrogen) following manufacturer’s protocol. 500 ng of total RNA was treated with DNAse I (Invitrogen), and subsequently used to generate cDNA. qRT-PCR was performed using SYBR Green (Roche) on a Lightcycler 480 system II (Roche). In case of multi-tissue qPCRs, the geometric mean of multiple reference genes was used [32]. Analysis of qRT-PCR data was done using LinRegPCR analysis software [33]. End-point RT-PCR was performed with Hot Fire Taq polymerase (Solis Biodyne) according to a standard protocol. All primer sequences can be found in S1 Table.

### Protein isolation and Western blotting

Protein was isolated in RIPA buffer (50mM Tris-HCl pH8, 150mM NaCl, 1% NP-40, 0.2% sodium deoxycholate, 0.1% SDS, 1 mM Na3VO4, 1 mM PMSF) supplemented with protease inhibitor cocktail (Roche). Protein concentration was measured using a BCA assay (Pierce). Western blotting was performed following standard protocols. Proteins were resolved by SDS-PAGE, transferred to PVDF membranes (Bio-Rad), and incubated with primary antibodies overnight at 4°C. Membranes were subsequently incubated with HRP-conjugated secondary antibodies for 1 hour at room temperature. Western blots were developed with ECL prime western blotting detection agent (Amersham Biosciences). Western blots were quantified using AIDA Image Analyzer v4.26 software. Antibodies used can be found in S1 Table.

### Histological analysis

Histological analysis was carried out as previously described [29]. Mouse hearts were fixed overnight in 4% paraformaldehyde, transferred to 70% ethanol, dehydrated, and embedded in paraffin using standard techniques. Sections of 5 μm thickness were stained with hematoxylin and eosin, or picrosirius red. To quantify the amount of fibrosis, 5 pictures of the Sirius Red stained sections per LV were taken with a light microscope (20x magnification). From these pictures, the Sirius Red positive area was automatically calculated as a percentage of the total tissue area using an in-house made quantification macro in ImagePro 6.2. This macro provided the total amount of tissue pixels and the Sirius Red positive pixels per picture by manual threshold settings. Perivascular fibrosis was manually omitted from the pictures.

### Statistical analysis

Data are presented as mean ± sem, and were analyzed with appropriate statistical tests, as indicated in the respective figure legend. A value of p < 0.05 was considered statistically significant.

## Results

### Rbm38 mRNA is ubiquitously expressed

**Fig 1:**
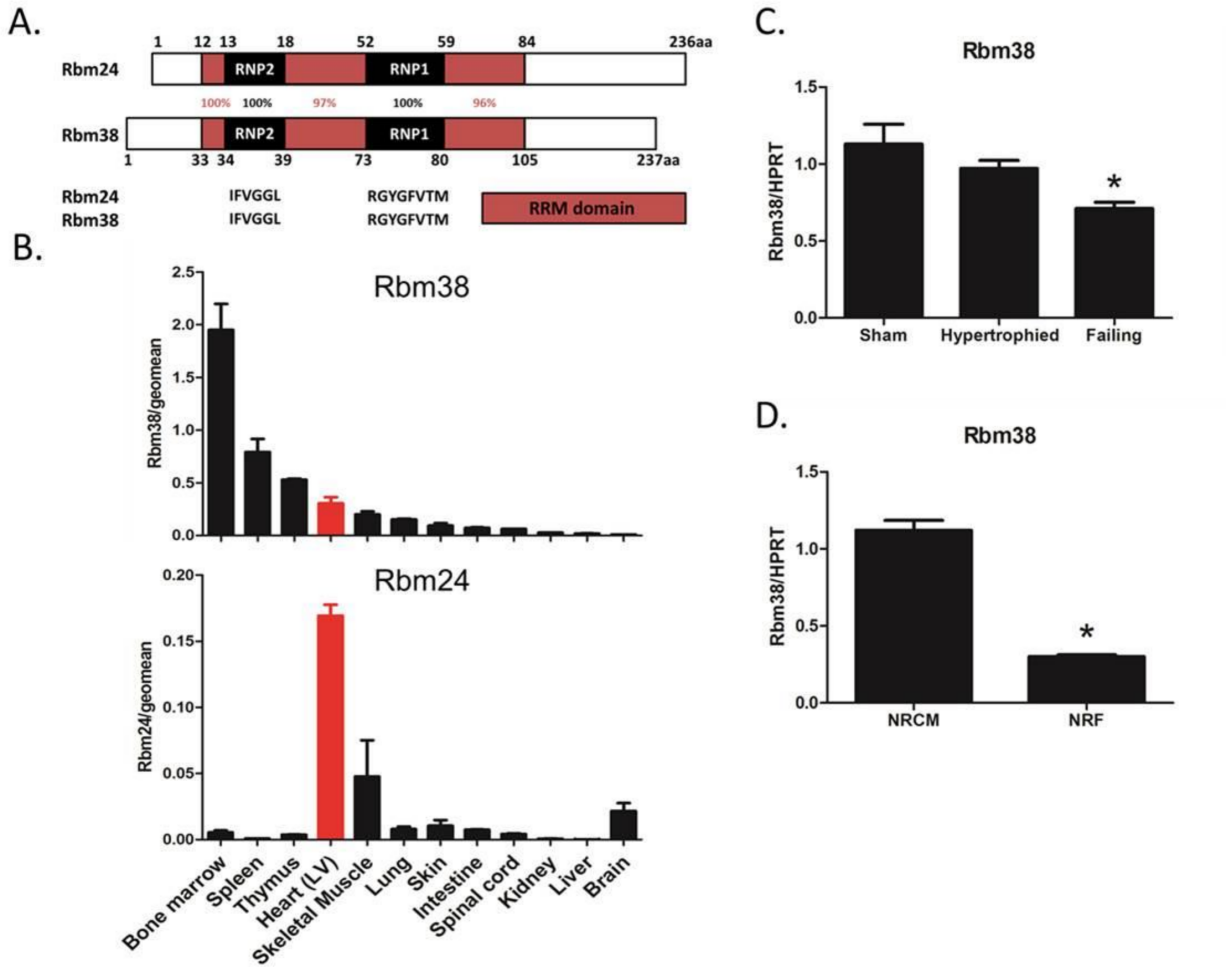
Rbm38 is ubiquitously expressed. A. Sequence identity of mouse Rbm24 and Rbm38. RRM represents the RNA-recognition motif, including the submotifs RNP1 and RNP2. B. qPCR of Rbm24 and Rbm38 in mouse tissues. Left ventricular (LV) heart tissue is highlighted in red. Values are corrected for the geometric mean of the following reference genes: Gapdh, Hprt, Pgk1, Rpl32, and Tbp. C. qPCR of Rbm38 in sham-operated and TAC-operated wildtype mice (C57/Bl6) 12 weeks after surgery. Hypertrophied mice were TAC-operated mice with an ejection fraction > 35%, failing mice were TAC-operated mice with an ejection fraction < 20%. D. qPCR of Rbm38 in neonatal rat cardiomyocytes (NRCM) and neonatal rat fibroblasts (NRF). Significance was tested with a 2-tailed Student’s t-test, * indicates p < 0.05 versus sham.

Rbm38, a known homolog of the heart- and muscle- specific splice factor Rbm24, shares 68% sequence identity at the amino acid level with Rbm24 (Fig 1A). Within the RNA recognition motif (RRM) the homology is even 97%, suggesting Rbm38 is able to bind similar RNA sequences as Rbm24. In order to investigate the expression pattern of Rbm38 and Rbm24, we performed quantitative PCR (qPCR) on cDNA derived from a wide range of human and mouse tissues. We found Rbm38 to be ubiquitously expressed, with the most abundant expression in hematopoietic tissues such as the spleen and bone marrow, and moderate expression in other tissues such as heart- and skeletal muscle (mouse tissue panel in Fig 1B, human tissue panel in S1 Fig). In line with previous studies, we detected Rbm24 mainly in heart- and skeletal muscle (Fig 1B and S1 Fig) [16, 34]. In wildtype C57/Bl6 mice, we found a decrease of Rbm38 mRNA 12 weeks after TAC (ejection fraction of failing hearts < 20%, ejection fraction of hypertrophied hearts > 35%) (Fig 1C). With respect to cellular expression within the heart, we found Rbm38 to be expressed in both cardiomyocytes and fibroblasts, as determined by qPCR on isolated neonatal rat cardiomyocytes (NRCM) and neonatal rat fibroblasts (NRF) (Fig 1D). Considering the homology with the pivotal cardiac splice factor Rbm24, we hypothesized that Rbm38 plays a role in cardiac remodeling.

### Generation of Rbm38 knockout mice

**Fig 2:**
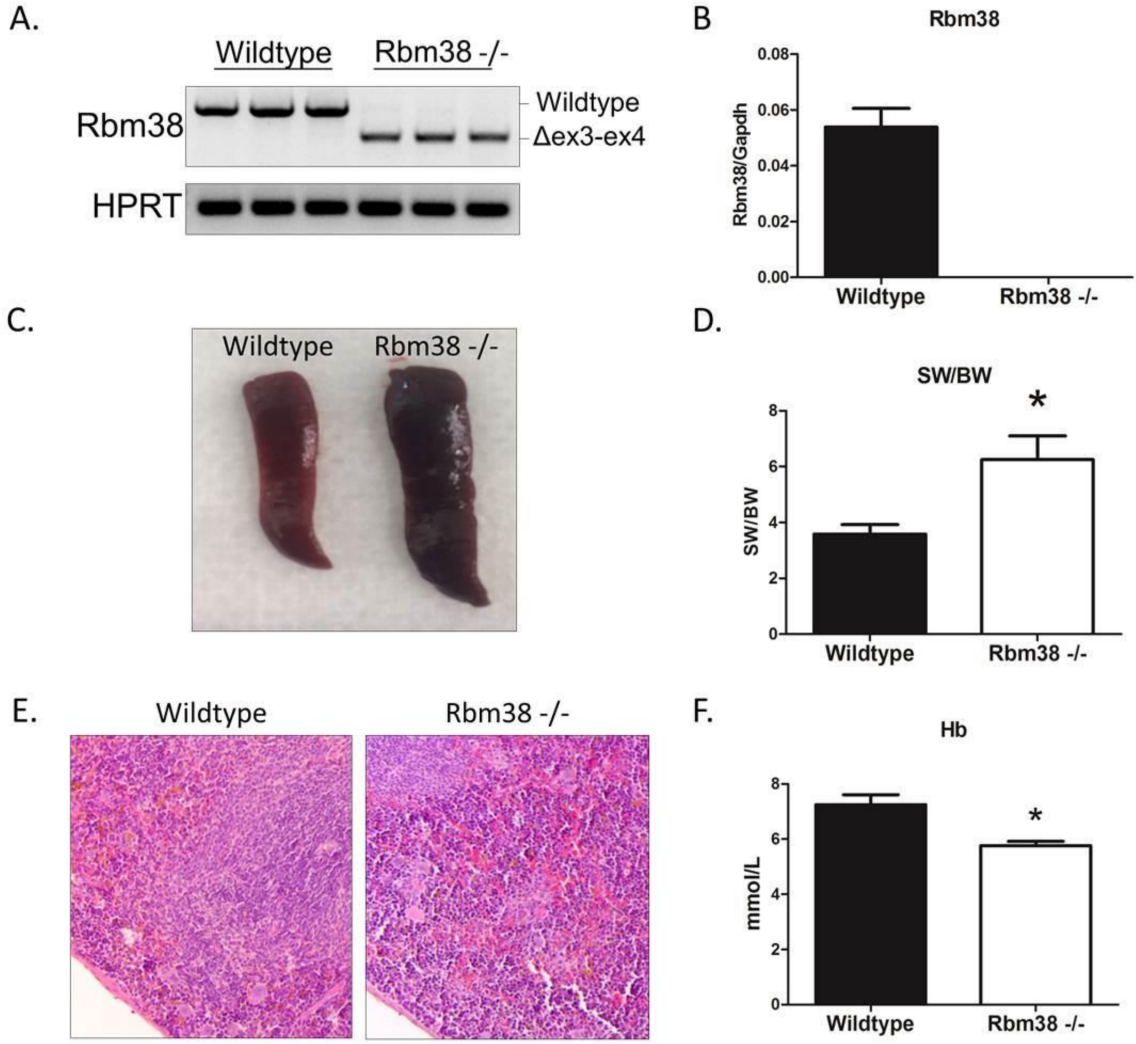
Rbm38 knockout mice have hematopoietic defects. A. RT-PCR of Rbm38 mRNA transcript in wildtype and Rbm38 -/- hearts. Δex3-4 represents the transcript lacking exon 3 and 4. B. qPCR of wildtype Rbm38 mRNA with primers designed within exon 4 and 5. C. Representative photograph of a wildtype and Rbm38 -/- spleen of 15–18 weeks old mice. D. Spleen weight/body weight ratio of 15–18 weeks old wildtype (n = 7) and Rbm38 -/- mice (n = 3). E. Representative images of H&E staining on section of wildtype and Rbm38 -/- spleens. Note the increased number of nucleated red blood cells in the splenic red pulp indicative of extramedullary hematopoiesis (EMH). F. Hemoglobin levels in the blood of wildtype (n = 7) and Rbm38 -/- mice (n = 5). Significance was tested with a 2-tailed Student’s t-test, * indicates p < 0.05 versus wildtype.

To study the function of Rbm38 in the heart, we generated Rbm38-deficient mice by targeted disruption of exon 3 and 4 of the Rbm38 gene. The mouse Rbm38 gene encompasses 5 exons and spans ~ 13 kb of genomic DNA. The open reading frame of Rbm38 starts in exon 2, and the RNA recognition motif is located in exons 2 and 3. The deletion of exons 3 and 4 is predicted to result in a premature stop codon, which would either lead to nonsense-mediated decay of the mRNA or the production of a truncated protein (108 amino acids) with a disrupted RNA-binding domain. As shown in Fig 2A, Rbm38 -/- mice were successfully targeted, as they express an Rbm38 transcript that lacks exon 3 and 4, which was confirmed by Sanger sequencing (S2A Fig). We also detected a transcript with a splice junction from an alternative 5’ splice site in exon 2 (23 bp upstream of the conventional 5’ splice site) to exon 5. This transcript is predicted to produce a truncated protein of 70 amino acids (S2B Fig). The expression level of these shorter transcripts was slightly lower than the wildtype transcript, suggestive of some level of nonsense mediated decay. The wildtype Rbm38 transcript was undetectable in the hearts of Rbm38 -/- mice, as qRT-primers designed within exon 4 and 5 did not amplify any product in the Rbm38 -/- hearts (Fig 2B).

### Rbm38 knockout mice have hematopoietic defects

Mice homozygous for the mutant Rbm38 allele were born in normal Mendelian ratios, were viable and did not show overt abnormalities. However, at autopsy we noticed that the spleens of Rbm38 -/- mice were enlarged and measuring the spleen weight/body weight ratio revealed that Rbm38 -/- spleens were almost 2-fold heavier than wildtype spleens (Fig 2C and 2D). H&E staining of the spleens of wildtype and Rbm38 -/- mice revealed extensive extramedullary hematopoiesis (EMH) as evidenced by increased numbers of nucleated red blood cells (Fig 2E). Blood analysis revealed decreased hemoglobin levels, indicating that Rbm38 -/- mice are anemic (Fig 2F). This suggests that the enlarged spleen with EMH is likely a compensatory mechanism for the anemia seen in the Rbm38 -/- mice. Overall, these data recapitulate the hematopoietic defects seen in the recently published Rbm38 -/- mouse model [20].

### Cardiac performance of Rbm38 knockout mice

We used M-mode echocardiography to functionally characterize the hearts of the Rbm38 -/- mice at 8–11 weeks of age (Table 1). Interestingly, left ventricular anterior wall thickness (LVAW) during systole was significantly increased in the Rbm38 -/- hearts compared to wildtype hearts (1.51±0.04 versus 1.39±0.03, p = 0.03). We also found increased fractional shortening (FS) in Rbm38-/- mice, but these data did not reach statistical significance (30.3±1.0 versus 28.2±0.6, p = 0.06). This indicates that the hearts of Rbm38 -/- mice were moderately hypertrophied, with a potentially small positive effect on cardiac function.

**Table 1:** Echocardiography of 8–11 weeks old wildtype and Rbm38 -/- mice.

	Wildtype (n = 15)	Rbm38 -/- (n = 13)	p-value
**LVID;d**	**3.94 ± 0.04**	**3.89 ± 0.04**	**0.46**
**LVID;s**	**2.83 ± 0.04**	**2.71 ± 0.07**	**0.07**
**LVAW;d**	**1.04 ± 0.03**	**1.11 ± 0.13**	**0.13**
**LVAW;s**	**1.39 ± 0.03**	**1.51 ± 0.04**	**0.03**
**LVPW;d**	**0.74 ± 0.03**	**0.77 ± 0.03**	**0.40**
**LVPW;s**	**1.00 ± 0.03**	**1.01 ± 0.03**	**0.84**
**%FS**	**28.21 ± 0.59**	**30.34 ± 0.96**	**0.06**
**%EF**	**55.06 ± 0.91**	**58.20 ± 1.37**	**0.06**

LVID: left ventricular internal diameter, LVAW: left ventricular anterior wall thickness, LVPW: left ventricular posterior wall thickness, %FS: percentage fractional shortening, %EF: percentage ejection fraction, ;d is during diastole, and ;s is during systole. Data are presented as mean ± sem, and were tested with a 2-tailed Student’s t-test.

### Rbm38 is dispensable during pressure overload in the mouse heart

**Fig 3:**
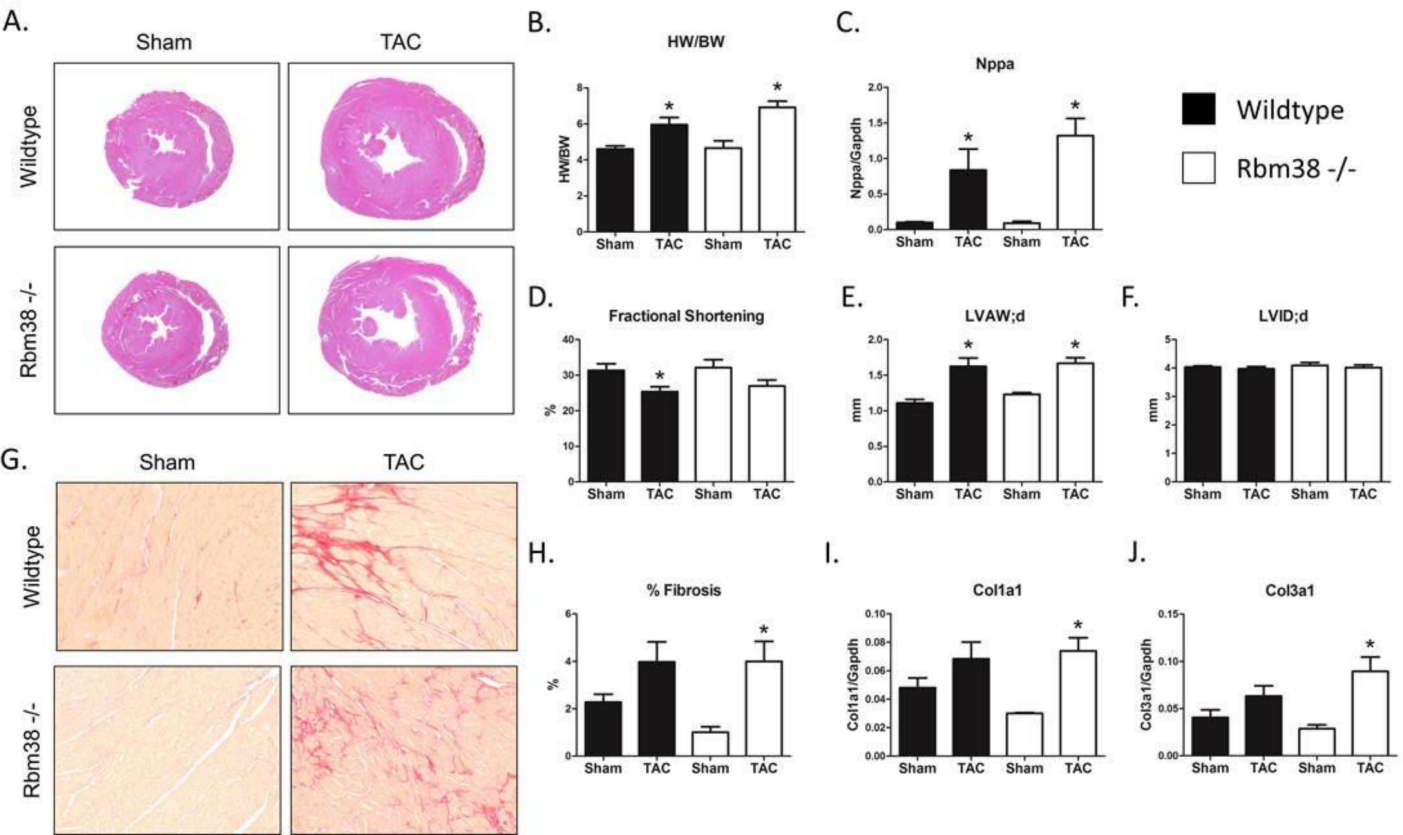
Rbm38 is dispensable during pressure overload-induced cardiac remodeling. A. Representative images of H&E stained sections of wildtype and Rbm38 -/- hearts 7 weeks after sham or TAC surgery. B. Heart weight/body weight ratios of wildtype and Rbm38 -/- mice C. ANF levels were measured by qPCR in wildtype and Rbm38 -/- mice. D-F. Echocardiographic parameters in wildtype and Rbm38 -/- mice G. Representative images of Picrosirius red stained sections of wildtype and Rbm38 -/- hearts H. Quantification of fibrosis. I-J. Col1a1 and Col3a1 mRNA levels measured by qPCR. Number of animals per group: wildtype sham n = 7, wildtype TAC n = 8, Rbm38 -/- sham n = 3, Rbm38 -/- TAC n = 7. Statistical significance was tested by a 1-way ANOVA with LSD posthoc-testing, * indicates p < 0.05 versus sham.

To uncover a potential role of Rbm38 during pressure overload-induced cardiac remodeling, we subjected Rbm38 -/- and wildtype littermates to 7 weeks of TAC. While pressure overload increased cardiac dimensions in both genotypes, histological analysis revealed no gross morphological differences between wildtype and Rbm38 -/- hearts (Fig 3A). In addition, both wildtype and Rbm38 -/- mice showed a significant increase in heart weight/body weight ratio (HW/BW), and a trend towards increased lung weight/body weight ratio (LW/BW) upon TAC (Fig 3B and S3 Fig). 2-way ANOVA did not reveal statistically significant interaction between genotype and TAC intervention. Furthermore, the stress marker atrial natriuretic peptide (Nppa) was induced to a similar extent in wildtype and Rbm38 -/- hearts after TAC (Fig 3C). Echocardiographic analysis showed similar increases in wall thickness (LVAW;d) and similar decreases in fractional shortening in response to TAC in wildtype and Rbm38 -/- mice (Fig 3D and 3E). Left ventricular internal diameter (LVID) was not increased in response to TAC, neither did we observe differences between wildtype or Rbm38 -/- mice (Fig 3F). Picrosirius red staining of sections of wildtype and Rbm38 -/- hearts revealed no differences in the amount of fibrosis after TAC (Fig 3G and 3H). Even though we saw a trend towards decreased fibrosis at baseline in the hearts of Rbm38 -/- mice, both in histological analysis and at mRNA expression level of Col1a1 and Col3a1 (Fig 3H–3J), this difference did not reach statistical significance. In conclusion, cardiac remodeling progressed independent of Rbm38, as we found no significant differences between wildtype and Rbm38 -/- mice after TAC.

### Regulation of previously identified Rbm38 targets in Rbm38 -/- hearts

**Fig 4:**
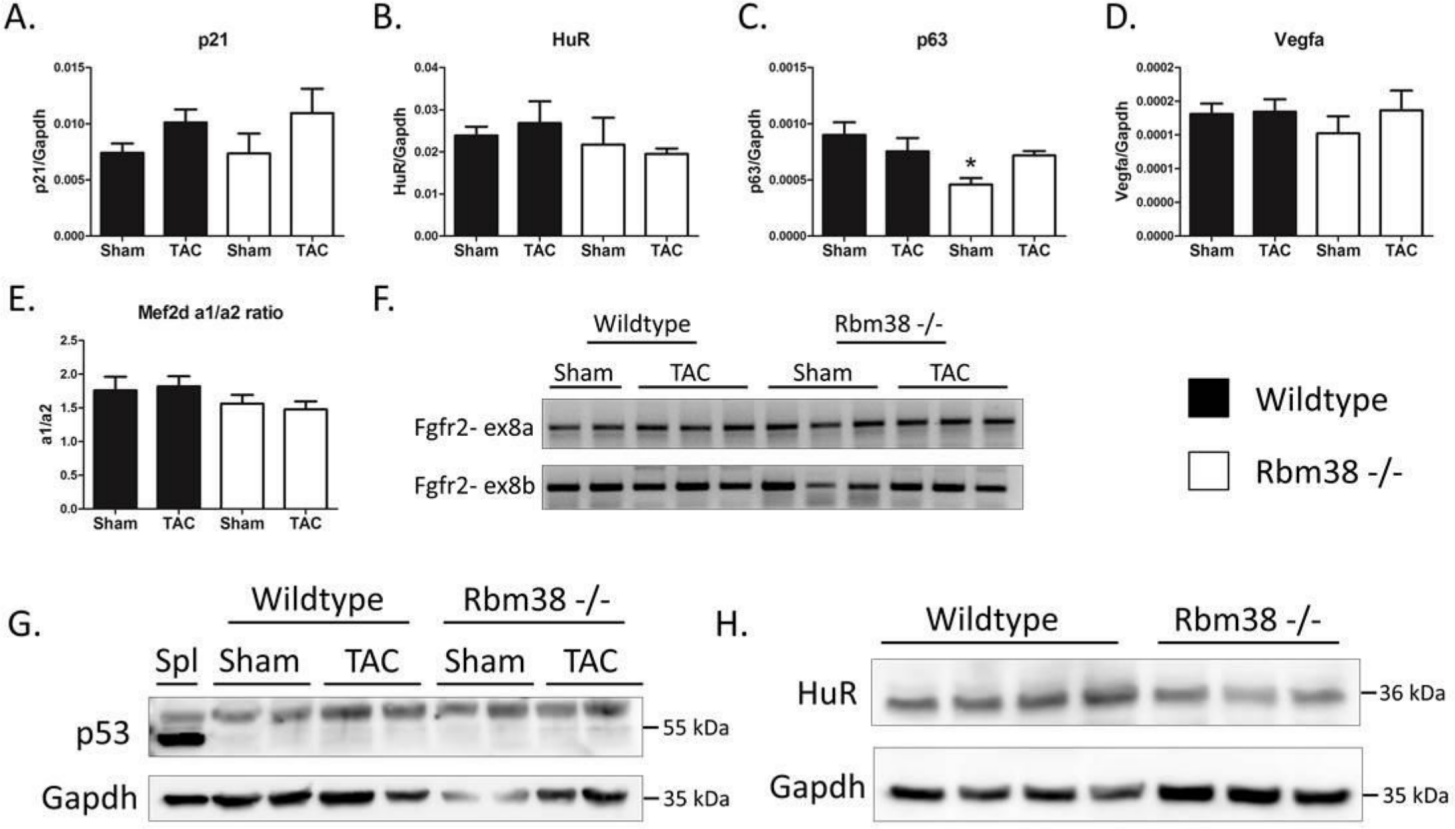
Regulation of previously identified Rbm38 targets. A-D. qPCR analysis of previously identified Rbm38 targets in hearts of wildtype and Rbm38 -/- mice. E. qPCR analysis of Mef2d exon a1 or a2 inclusion. F. RT-PCR of Fgfr2 exon 8a or 8b inclusion. G. Western blot of p53 in wildtype versus Rbm38 -/- hearts. Spleen (Spl) serves as a positive control for p53 protein expression. H. Western blot of HuR in wildtype versus Rbm38 -/- hearts. Statistical significance was tested by a 1-way ANOVA with LSD posthoc-testing, * means p < 0.05 versus wildtype.

We next asked whether the loss of Rbm38 in the heart would affect previously identified targets of Rbm38. First, we analyzed the RNA stability targets p21, p63, and HuR in the hearts of Rbm38 -/- mice [10, 11, 17], but we found no regulation of p21 and HuR mRNA (Fig 4A and 4B). We did find downregulation of p63 mRNA in Rbm38 -/- sham hearts compared to wildtype sham hearts, which is contradictory to what we expected (Fig 4C). In this regard, Rbm38 has been shown to destabilize the p63 transcript, so upon loss of Rbm38 we expected to find an increase in p63 mRNA levels. Hypoxia inducable factor 1α (Hif1α) is another stability target gene of Rbm38 in the breast- and colon carcinoma cell lines MCF7 and HCT116 [35]. We therefore examined the expression of one of the direct Hif1α-target genes, Vegfa. As shown in Fig 4D, we found no differences in Vegfa expression, suggesting Hif1α was not regulated in the Rbm38 -/- hearts. To investigate the splicing potential of Rbm38 in the heart we performed (q)RT-PCRs for the previously identified Rbm38 splicing targets myocyte enhancer factor 2D (Mef2d) and Fgfr2 [14, 19]. We quantified the ratio of exon α1/α2 inclusion in Mef2d using qPCR, and performed RT-PCR to investigate exon 8a/8b inclusion in Fgfr2 and found no evidence of splicing changes of these genes in the hearts of Rbm38 -/- mice (Fig 4E and 4F). Next, we investigated some of these Rbm38 targets in the spleens of Rbm38 -/- mice. We first examined Rbm38 expression in Rbm38 -/- spleens, and as expected the Rbm38 trancript was undetectable in Rbm38 -/- spleens (S4A Fig). We then analyzed the stability targets HuR and p21, but did not find regulation of HuR mRNA. We did, however, find a striking upregulation of p21 mRNA (S4B and S4C Fig). Rbm38 is known to stabilize p21, so the upregulation of p21 is opposite to what was expected upon loss of Rbm38. The splicing target Mef2d, which was identified in red blood cells [19], seemed to be affected in Rbm38 -/- spleens, as we found a decrease in the exon α1/α2 inclusion ratio (p = 0.07) (S4D Fig). It is conceivable that splicing differences in Mef2d were only found in Rbm38 -/- spleens, and not in Rbm38 -/- hearts, due to the large number of maturing red blood cells in this organ. Lastly, we examined toll-like receptor 7 (TLR7), an Rbm38 target identified in Rbm38 -/- mouse embryonic fibroblasts (MEFs) [20], in the spleens and hearts of Rbm38 -/- mice. Although TLR7 was upregulated in Rbm38 -/- MEFs, we could not recapitulate this finding in either Rbm38 -/- spleens or hearts (S4E and S4F Fig).

We next wondered whether targets of Rbm24 could be affected, as Rbm24 and Rbm38 are highly alike, but we observed no differences in the known splicing targets of Rbm24 Coro6 and aNAC/skNAC (S5 Fig) [6]. We also examined the expression of p53 protein, a translational target of Rbm38 in lymphomas in Rbm38 -/- hearts [23]. Although we could readily detect p53 protein in the spleen, we were not able to detect p53 protein in the hearts of wildtype and Rbm38 -/- mice, neither in the healthy heart, nor after TAC surgery (Fig 4G). We also examined HuR protein levels in wildtype and Rbm38 -/- hearts, and even though we found no regulation on mRNA level, HuR protein levels were substantially decreased in the hearts of Rbm38-/- mice (Fig 4H and S6 Fig).

### Loss of Rbm38 might be functionally compensated by Rbm24

**Fig 5:**
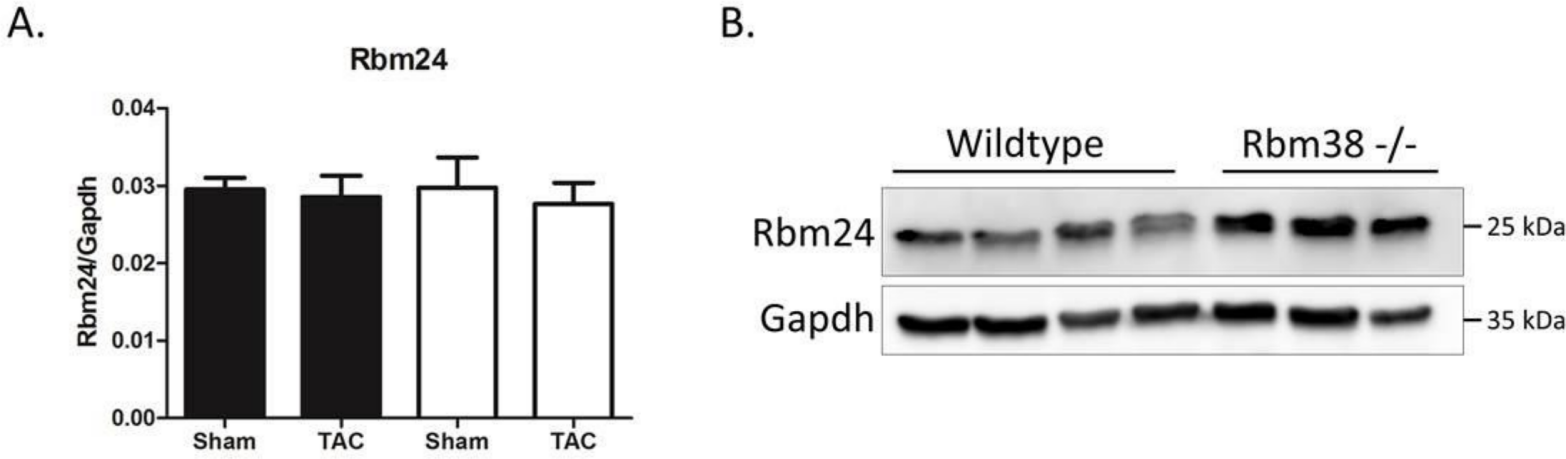
Rbm24 is upregulated in Rbm38 -/- hearts. A. Rbm24 mRNA levels were measured by qPCR. B. Western blot of Rbm24 in wildtype versus Rbm38 -/- hearts. Statistical significance was tested by a 1-way ANOVA with LSD posthoc-testing.

Since most previously reported target genes of Rbm38 do not show any differences in the hearts of Rbm38 -/- mice, we investigated whether redundancy with Rbm24 may have accounted for the lack of target regulation. Therefore, we performed qPCR and Western blotting of Rbm24, the closely related family member of Rbm38. Interestingly, we found no difference in Rbm24 mRNA levels, but we did see a trend of increased Rbm24 protein in Rbm38-/- hearts (p = 0.08) (Fig 5A and 5B and S6 Fig). This suggests that Rbm24 might functionally compensate for the loss of Rbm38 in Rbm38 -/- hearts, but additional studies are needed to test this hypothesis.

## Discussion

Here, we investigated the role of Rbm38 in the healthy and diseased heart. We found that Rbm38 is readily expressed in the heart, but that it is expressed most abundantly in hematopoietic tissues such as the spleen and bone marrow. We generated somatic Rbm38 -/- mice and found that Rbm38 -/- mice have hematopoietic defects, thereby recapitulating the anemic phenotype seen in a previously reported Rbm38 -/- mouse model [20]. The hearts of Rbm38 -/- mice were moderately hypertrophied, but echocardiography revealed that cardiac function was not compromised at 8–11 weeks of age. We did not find regulation of the previously identified stability target genes of Rbm38 p21 and HuR, neither did we find differences in splicing of Mef2d or Fgfr2. This lack of regulation can potentially be attributed to a redundancy mechanism, as the closely related RBP Rbm24 with which Rbm38 shares several targets, was found to be increased in Rbm38 -/- hearts.

TAC studies revealed that the hypertrophic response is slightly increased in Rbm38-/- mice. However, since Rbm38 -/- hearts are already hypertrophic at baseline, this trend can be secondary to the observed anemia in these mice. Overall, we conclude there is no vital role for Rbm38 in the heart, but a more subtle role cannot be excluded. In that sense, it would be interesting to use a cardiomyocyte-specific Rbm38 knockout mouse model, thereby circumventing the hematopoietic disorders, to dissect the role of Rbm38 specifically in cardiomyocytes. Also, to further examine the functional redundancy between Rbm38 and Rbm24, it would be interesting to generate compound Rbm24/Rbm38 knockout mice and investigate the pathophysiology in these hearts. Considering the high sequence identity between Rbm24 and Rbm38, it is conceivable that introducing one mutant Rbm24 allele into the Rbm38 null background would lead to abberant splicing and sarcomeric dysfunction, in line with the cardiac phenotype of the Rbm24 null mice [6]. However, although Rbm24 might compensate for the loss of Rbm38 in Rbm38 -/- mice, vice versa this might not be the case, as the Rbm24 knockout is embryonically lethal while the Rbm38 knockout is not [6, 20]. This could be due to differences in temporal expression of Rbm24 and Rbm38, as Rbm24 seems to be expressed mostly during embryonic stages, while Rbm38 seems to be expressed at the perinatal and adult stage.

The overlap in targets between Rbm24 and Rbm38 is likewise only partial, and it seems that most of the overlap occurs in genes involved in the p53-axis, such as p21 and p63 [7–11]. Despite previous reports showing that the p21 mRNA transcript is stabilized by both Rbm24 and Rbm38 in breast- and colon carcinoma cell lines [7, 9, 10], we did not observe reduced p21 mRNA levels in Rbm38 -/- hearts. This may be due to the increase in Rbm24 protein levels that we observed in the Rbm38 -/- heart, as this may have compensated for the loss of Rbm38. Remarkably, in Rbm38 -/- spleens, we found an upregulation of p21 mRNA, which is opposite to what was expected as it is previously reported that p21 mRNA is stabilized by Rbm38. In the spleen, however, compensation by Rbm24 is not likely, as Rbm24 is not expressed in the spleen. This indicates that p21 does not only rely on Rbm38 and/or Rbm24, but that the regulation of p21 is much more complex, especially *in vivo*. p63 mRNA, on the other hand, was previously shown to be destabilized by Rbm38 and Rbm24 [8, 11], and loss of Rbm38 in the heart is therefore expected to result in elevated p63 mRNA levels. We found, however, that p63 mRNA was significantly downregulated in Rbm38-/- hearts. This could be due to the expressed isoform of p63, as it has been recently shown that the direction of regulatory effect of Rbm38 also depends on the isoform of p63 [36]. Whether this lack of effect or contradictory effect on these targets are due to increased Rbm24 levels in the Rbm38-/- heart, or whether the exact mechanisms of target regulation by Rbm38 are tissue-specific remains elusive. We were not able to detect the translational target p53 in the hearts of wildtype and Rbm38 -/- mice, both before and after TAC surgery. Interestingly, p53 has been described to be upregulated during pressure overload-induced cardiac remodeling, where it inhibits Hif1a activity, and aggravates maladaptive hypertrophy through inhibition of the angiogenic response in the heart [27]. However, using two independent antibodies against p53 we were not able to replicate this finding. We also did not find reduced expression of Vegfa, a direct target gene of Hif1a, suggesting that Hif1a-signaling was not affected. Under hypoxic conditions Hif1a itself, however, is also a direct translational target of Rbm38, and the loss of Rbm38 could therefore have increased Hif1a protein levels [35]. Considering the lack of effect on Vegfa, this does not seem to be the case in Rbm38 -/- hearts. Overall, we conclude that the targets of Rbm38 are highly tissue- or cell-type specific, and potentially relies on tissue- or cell-type specific co-factors. Therefore, it will be interesting to uncover the regulatory capacity of Rbm38 in different tissues.

The role of anemia in the development of cardiac hypertrophy and failure is not entirely clear, but it seems that anemia can induce a mild hypertrophic phenotype, similar to what we observe in our mouse model [37]. Recent studies have shown that anemia is not involved in the progression of heart failure [37, 38]. For example, in an experimental mouse model of chronic renal failure, mice develop anemia and cardiac dysfunction. In these mice, activating erythropoietin (EPO) signaling is cardioprotective, but this intervention is unrelated to the relief of anemia [37]. Interestingly, when EPO signaling is activated using recombinant EPO, cardiac dysfunction is diminished and anemia is relieved through stimulation of erythropoiesis. When asialoEPO, an EPO derivative that does not affect erythropoiesis, is used, anemia is not relieved, but cardiac dysfunction is likewise diminished [37]. This indicates that anemia is not involved in the progression of cardiac remodeling and failure, but can induce a mild hypertrophic heart.

In summary, we show that loss of Rbm38 leads to hematopoietic defects. In the heart, loss of Rbm38 leads to downregulation of HuR protein levels, but we found most previously published Rbm38 targets to be unaffected. However, most of these targets were identified in different cells or tissues, which could mean that these are cell- or tissue-specific. In addition, we find the closely related RBP Rbm24 to be increased in Rbm38 -/- hearts, which suggests redundancy between Rbm24 and Rbm38 exists. Overall, we propose that Rbm38 does not play an important role in the normal and diseased heart, but is more critical in erythroid differentiation.

## Supporting information

S1 Fig RBM38 and RBM24 mRNA expression in human tissue panel. A. qPCR of RBM24 and RBM38 in human RNA tissue panel. Values are corrected for the geometric mean of the following reference genes: GAPDH, HPRT, and B2M.
(PDF)

S2 Fig Sequence of the targeted Rbm38 mRNA transcript. PCR product of Rbm38 -/- hearts in Fig 2A was cloned into pGEM T-easy and 8 clones were Sanger sequenced. A. 7 out of 8 clones showed a splice junction from exon 2 to exon 5 resulting in a transcript that is predicted to produce a protein of 108 amino acids. B. 1 of the 8 clones contained a splice junction of an alternative splice site in exon 2 (23 bp before the end of exon 2) to exon 5, resulting in a transcript that is predicted to produce a protein of 70 amino acids. Dotted red line indicates the splice junction.
(PDF)

S3 Fig Lung weight/Body weight ratio after TAC. Lung weight/Body weight ratio in sham-operated and TAC-operated wildtype and Rbm38 -/- mice 7 weeks after surgery.
(PDF)

S4 Fig Rbm38 targets in Rbm38 -/- spleens and hearts. A. qPCR analysis of Rbm38 in wildtype and Rbm38 -/- spleens. B. qPCR analysis of HuR in wildtype and Rbm38 -/- spleens. C. qPCR analysis of p21 in wildtype and Rbm38 -/- spleens. D. qPCR analysis of Mef2d exon α1/ α2 inclusion. E. qPCR analysis of TLR7 in wildtype and Rbm38 -/- spleens. F. qPCR analysis of TLR7 in wildtype and Rbm38 -/- hearts. Wildtype spleens n = 5, Rbm38 -/- spleens n = 5. Wildtype hearts n = 5, Rbm38 -/- hearts n = 3.
(PDF)

S5 Fig Splicing of Rbm24 targets is unaltered in Rbm38 -/- hearts. RT-PCR of Rbm24 splicing targets Coro6, aNAC, and skNAC. HPRT was used as a loading control.
(PDF)

S6 Fig Quantification of Western blots in Fig 5. A. Quantification of Western blot in Fig 4H. B. Quantification of Western blot in Fig 5B.
(PDF)

S1 Table List of primers and antibodies used. (XLS)

## References

[1] van den Hoogenhof MM, Pinto YM, Creemers EE. RNA Splicing: Regulation and Dysregulation in the Heart. Circ Res. 2016;118(3):454–68. doi: 10.1161/CIRCRESAHA.115.307872 . 26846640 10.1161/CIRCRESAHA.115.307872

[2] Weeland CJ, van den Hoogenhof MM, Beqqali A, Creemers EE. Insights into alternative splicing of sarcomeric genes in the heart. J Mol Cell Cardiol. 2015;81:107–13. doi: 10.1016/j.yjmcc.2015.02.008 . 25683494 10.1016/j.yjmcc.2015.02.008

[3] Brauch KM, Karst ML, Herron KJ, de Andrade M, Pellikka PA, Rodeheffer RJ, et al Mutations in ribonucleic acid binding protein gene cause familial dilated cardiomyopathy. J Am Coll Cardiol. 2009;54(10):930–41. doi: 10.1016/j.jacc.2009.05.038 ; PubMed Central PMCID: PMCPMC2782634. 19712804 10.1016/j.jacc.2009.05.038 PMC2782634

[4] Li D, Morales A, Gonzalez-Quintana J, Norton N, Siegfried JD, Hofmeyer M, et al Identification of novel mutations in RBM20 in patients with dilated cardiomyopathy. Clin Transl Sci. 2010;3(3):90–7. doi: 10.1111/j.1752-8062.2010.00198.x ; PubMed Central PMCID: PMCPMC2898174. 20590677 10.1111/j.1752-8062.2010.00198.x PMC2898174

[5] Beqqali A, Bollen IA, Rasmussen TB, van den Hoogenhof MM, van Deutekom HW, Schafer S, et al A mutation in the glutamate-rich region of RNA-binding motif protein 20 causes dilated cardiomyopathy through missplicing of titin and impaired Frank-Starling mechanism. Cardiovasc Res. 2016;112(1):452–63. doi: 10.1093/cvr/cvw192 . 27496873 10.1093/cvr/cvw192

[6] Yang J, Hung LH, Licht T, Kostin S, Looso M, Khrameeva E, et al RBM24 is a major regulator of muscle-specific alternative splicing. Dev Cell. 2014;31(1):87–99. doi: 10.1016/j.devcel.2014.08.025 . 25313962 10.1016/j.devcel.2014.08.025

[7] Jiang Y, Zhang M, Qian Y, Xu E, Zhang J, Chen X. Rbm24, an RNA-binding protein and a target of p53, regulates p21 expression via mRNA stability. J Biol Chem. 2014;289(6):3164–75. doi: 10.1074/jbc.M113.524413 ; PubMed Central PMCID: PMCPMC3916521. 24356969 10.1074/jbc.M113.524413 PMC3916521

[8] Xu E, Zhang J, Zhang M, Jiang Y, Cho SJ, Chen X. RNA-binding protein RBM24 regulates p63 expression via mRNA stability. Mol Cancer Res. 2014;12(3):359–69. doi: 10.1158/1541-7786.MCR-13-0526 ; PubMed Central PMCID: PMCPMC3962715. 24375645 10.1158/1541-7786.MCR-13-0526 PMC3962715

[9] Cho SJ, Zhang J, Chen X. RNPC1 modulates the RNA-binding activity of, and cooperates with, HuR to regulate p21 mRNA stability. Nucleic Acids Res. 2010;38(7):2256–67. doi: 10.1093/nar/gkp1229 ; PubMed Central PMCID: PMCPMC2853136. 20064878 10.1093/nar/gkp1229 PMC2853136

[10] Shu L, Yan W, Chen X. RNPC1, an RNA-binding protein and a target of the p53 family, is required for maintaining the stability of the basal and stress-induced p21 transcript. Genes Dev. 2006;20(21):2961–72. doi: 10.1101/gad.1463306 ; PubMed Central PMCID: PMCPMC1620019. 17050675 10.1101/gad.1463306 PMC1620019

[11] Zhang J, Jun Cho S, Chen X. RNPC1, an RNA-binding protein and a target of the p53 family, regulates p63 expression through mRNA stability. Proc Natl Acad Sci U S A. 2010;107(21):9614–9. doi: 10.1073/pnas.0912594107 ; PubMed Central PMCID: PMCPMC2906842. 20457941 10.1073/pnas.0912594107 PMC2906842

[12] Anyanful A, Ono K, Johnsen RC, Ly H, Jensen V, Baillie DL, et al The RNA-binding protein SUP-12 controls muscle-specific splicing of the ADF/cofilin pre-mRNA in C. elegans. J Cell Biol. 2004;167(4):639–47. doi: 10.1083/jcb.200407085 ; PubMed Central PMCID: PMCPMC1781344. 15545320 10.1083/jcb.200407085 PMC1781344

[13] Kuroyanagi H, Ohno G, Mitani S, Hagiwara M. The Fox-1 family and SUP-12 coordinately regulate tissue-specific alternative splicing in vivo. Mol Cell Biol. 2007;27(24):8612–21. doi: 10.1128/MCB.01508-07 ; PubMed Central PMCID: PMCPMC2169414. 17923701 10.1128/MCB.01508-07 PMC2169414

[14] Warzecha CC, Sato TK, Nabet B, Hogenesch JB, Carstens RP. ESRP1 and ESRP2 are epithelial cell-type-specific regulators of FGFR2 splicing. Mol Cell. 2009;33(5):591–601. doi: 10.1016/j.molcel.2009.01.025 ; PubMed Central PMCID: PMCPMC2702247. 19285943 10.1016/j.molcel.2009.01.025 PMC2702247

[15] Alvarez-Dominguez JR, Zhang X, Hu W. Widespread and dynamic translational control of red blood cell development. Blood. 2016 doi: 10.1182/blood-2016-09-741835 . 27899360 10.1182/blood-2016-09-741835 PMC5290990

[16] Miyamoto S, Hidaka K, Jin D, Morisaki T. RNA-binding proteins Rbm38 and Rbm24 regulate myogenic differentiation via p21-dependent and -independent regulatory pathways. Genes Cells. 2009;14(11):1241–52. doi: 10.1111/j.1365-2443.2009.01347.x . 19817877 10.1111/j.1365-2443.2009.01347.x

[17] Cho SJ, Jung YS, Zhang J, Chen X. The RNA-binding protein RNPC1 stabilizes the mRNA encoding the RNA-binding protein HuR and cooperates with HuR to suppress cell proliferation. J Biol Chem. 2012;287(18):14535–44. doi: 10.1074/jbc.M111.326827 ; PubMed Central PMCID: PMCPMC3340227. 22371495 10.1074/jbc.M111.326827 PMC3340227

[18] Heinicke LA, Nabet B, Shen S, Jiang P, van Zalen S, Cieply B, et al The RNA binding protein RBM38 (RNPC1) regulates splicing during late erythroid differentiation. PLoS One. 2013;8(10):e78031 doi: 10.1371/journal.pone.0078031 ; PubMed Central PMCID: PMCPMC3820963. 24250749 10.1371/journal.pone.0078031 PMC3820963

[19] Ulirsch JC, Nandakumar SK, Wang L, Giani FC, Zhang X, Rogov P, et al Systematic Functional Dissection of Common Genetic Variation Affecting Red Blood Cell Traits. Cell. 2016;165(6):1530–45. doi: 10.1016/j.cell.2016.04.048 ; PubMed Central PMCID: PMCPMC4893171. 27259154 10.1016/j.cell.2016.04.048 PMC4893171

[20] Zhang J, Xu E, Ren C, Yan W, Zhang M, Chen M, et al Mice deficient in Rbm38, a target of the p53 family, are susceptible to accelerated aging and spontaneous tumors. Proc Natl Acad Sci U S A. 2014;111(52):18637–42. doi: 10.1073/pnas.1415607112 ; PubMed Central PMCID: PMCPMC4284600. 25512531 10.1073/pnas.1415607112 PMC4284600

[21] Leveille N, Elkon R, Davalos V, Manoharan V, Hollingworth D, Oude Vrielink J, et al Selective inhibition of microRNA accessibility by RBM38 is required for p53 activity. Nat Commun. 2011;2:513 doi: 10.1038/ncomms1519 ; PubMed Central PMCID: PMCPMC3221330. 22027593 10.1038/ncomms1519 PMC3221330

[22] Yan W, Zhang J, Zhang Y, Jung YS, Chen X. p73 expression is regulated by RNPC1, a target of the p53 family, via mRNA stability. Mol Cell Biol. 2012;32(13):2336–48. doi: 10.1128/MCB.00215-12 ; PubMed Central PMCID: PMCPMC3434491. 22508983 10.1128/MCB.00215-12 PMC3434491

[23] Zhang J, Cho SJ, Shu L, Yan W, Guerrero T, Kent M, et al Translational repression of p53 by RNPC1, a p53 target overexpressed in lymphomas. Genes Dev. 2011;25(14):1528–43. doi: 10.1101/gad.2069311 ; PubMed Central PMCID: PMCPMC3143942. 21764855 10.1101/gad.2069311 PMC3143942

[24] Zhang M, Zhang J, Chen X, Cho SJ, Chen X. Glycogen synthase kinase 3 promotes p53 mRNA translation via phosphorylation of RNPC1. Genes Dev. 2013;27(20):2246–58. doi: 10.1101/gad.221739.113 ; PubMed Central PMCID: PMCPMC3814645. 24142875 10.1101/gad.221739.113 PMC3814645

[25] Xue JQ, Xia TS, Liang XQ, Zhou W, Cheng L, Shi L, et al RNA-binding protein RNPC1: acting as a tumor suppressor in breast cancer. BMC Cancer. 2014;14:322 doi: 10.1186/1471-2407-14-322 ; PubMed Central PMCID: PMCPMC4101826. 24884756 10.1186/1471-2407-14-322 PMC4101826

[26] Carvalho B, Postma C, Mongera S, Hopmans E, Diskin S, van de Wiel MA, et al Multiple putative oncogenes at the chromosome 20q amplicon contribute to colorectal adenoma to carcinoma progression. Gut. 2009;58(1):79–89. doi: 10.1136/gut.2007.143065 . 18829976 10.1136/gut.2007.143065

[27] Sano M, Minamino T, Toko H, Miyauchi H, Orimo M, Qin Y, et al p53-induced inhibition of Hif-1 causes cardiac dysfunction during pressure overload. Nature. 2007;446(7134):444–8. doi: 10.1038/nature05602 . 17334357 10.1038/nature05602

[28] Skarnes WC, Rosen B, West AP, Koutsourakis M, Bushell W, Iyer V, et al A conditional knockout resource for the genome-wide study of mouse gene function. Nature. 2011;474(7351):337–42. doi: 10.1038/nature10163 ; PubMed Central PMCID: PMCPMC3572410. 21677750 10.1038/nature10163 PMC3572410

[29] Tijsen AJ, van der Made I, van den Hoogenhof MM, Wijnen WJ, van Deel ED, de Groot NE, et al The microRNA-15 family inhibits the TGFbeta-pathway in the heart. Cardiovasc Res. 2014;104(1):61–71. doi: 10.1093/cvr/cvu184 . 25103110 10.1093/cvr/cvu184

[30] Medzikovic L, Schumacher CA, Verkerk AO, van Deel ED, Wolswinkel R, van der Made I, et al Orphan nuclear receptor Nur77 affects cardiomyocyte calcium homeostasis and adverse cardiac remodelling. Sci Rep. 2015;5:15404 doi: 10.1038/srep15404 ; PubMed Central PMCID: PMCPMC4613907. 26486271 10.1038/srep15404 PMC4613907

[31] ter Welle HF, Baartscheer A, Fiolet JW, Schumacher CA. The cytoplasmic free energy of ATP hydrolysis in isolated rod-shaped rat ventricular myocytes. J Mol Cell Cardiol. 1988;20(5):435–41. . 3210251 10.1016/s0022-2828(88)80135-4

[32] Ruiz-Villalba A, Mattiotti A, Gunst QD, Cano-Ballesteros S, van den Hoff MJ, Ruijter JM. Reference genes for gene expression studies in the mouse heart. Sci Rep. 2017;7(1):24 doi: 10.1038/s41598-017-00043-9 . 28154421 10.1038/s41598-017-00043-9 PMC5428317

[33] Ruijter JM, Ramakers C, Hoogaars WM, Karlen Y, Bakker O, van den Hoff MJ, et al Amplification efficiency: linking baseline and bias in the analysis of quantitative PCR data. Nucleic Acids Res. 2009;37(6):e45 doi: 10.1093/nar/gkp045 ; PubMed Central PMCID: PMCPMC2665230. 19237396 10.1093/nar/gkp045 PMC2665230

[34] Poon KL, Tan KT, Wei YY, Ng CP, Colman A, Korzh V, et al RNA-binding protein RBM24 is required for sarcomere assembly and heart contractility. Cardiovasc Res. 2012;94(3):418–27. doi: 10.1093/cvr/cvs095 . 22345307 10.1093/cvr/cvs095

[35] Cho SJ, Teng IF, Zhang M, Yin T, Jung YS, Zhang J, et al Hypoxia-inducible factor 1 alpha is regulated by RBM38, a RNA-binding protein and a p53 family target, via mRNA translation. Oncotarget. 2015;6(1):305–16. doi: 10.18632/oncotarget.2786 ; PubMed Central PMCID: PMCPMC4381596. 25622105 10.18632/oncotarget.2786 PMC4381596

[36] Yan W, Zhang Y, Chen X. TAp63gamma and DeltaNp63gamma are regulated by RBM38 via mRNA stability and have an opposing function in growth suppression. Oncotarget. 2017 doi: 10.18632/oncotarget.18463 . 10.18632/oncotarget.18463 PMC5667965 29108232

[37] Ogino A, Takemura G, Kawasaki M, Tsujimoto A, Kanamori H, Li L, et al Erythropoietin receptor signaling mitigates renal dysfunction-associated heart failure by mechanisms unrelated to relief of anemia. J Am Coll Cardiol. 2010;56(23):1949–58. doi: 10.1016/j.jacc.2010.04.068 . 21109120 10.1016/j.jacc.2010.04.068

[38] Asaumi Y, Kagaya Y, Takeda M, Yamaguchi N, Tada H, Ito K, et al Protective role of endogenous erythropoietin system in nonhematopoietic cells against pressure overload-induced left ventricular dysfunction in mice. Circulation. 2007;115(15):2022–32. doi: 10.1161/CIRCULATIONAHA.106.659037 . 17404160 10.1161/CIRCULATIONAHA.106.659037

