# Supplementary figures and images for: The RNA-binding protein Rbm38 is dispensable during pressure overload-induced cardiac remodeling in mice
Source: PLoS One. 2017 Aug 29;12(8):e0184093. doi: 10.1371/journal.pone.0184093 (PMC5574583; doi:10.1371/journal.pone.0184093)

S1 Figure

A.

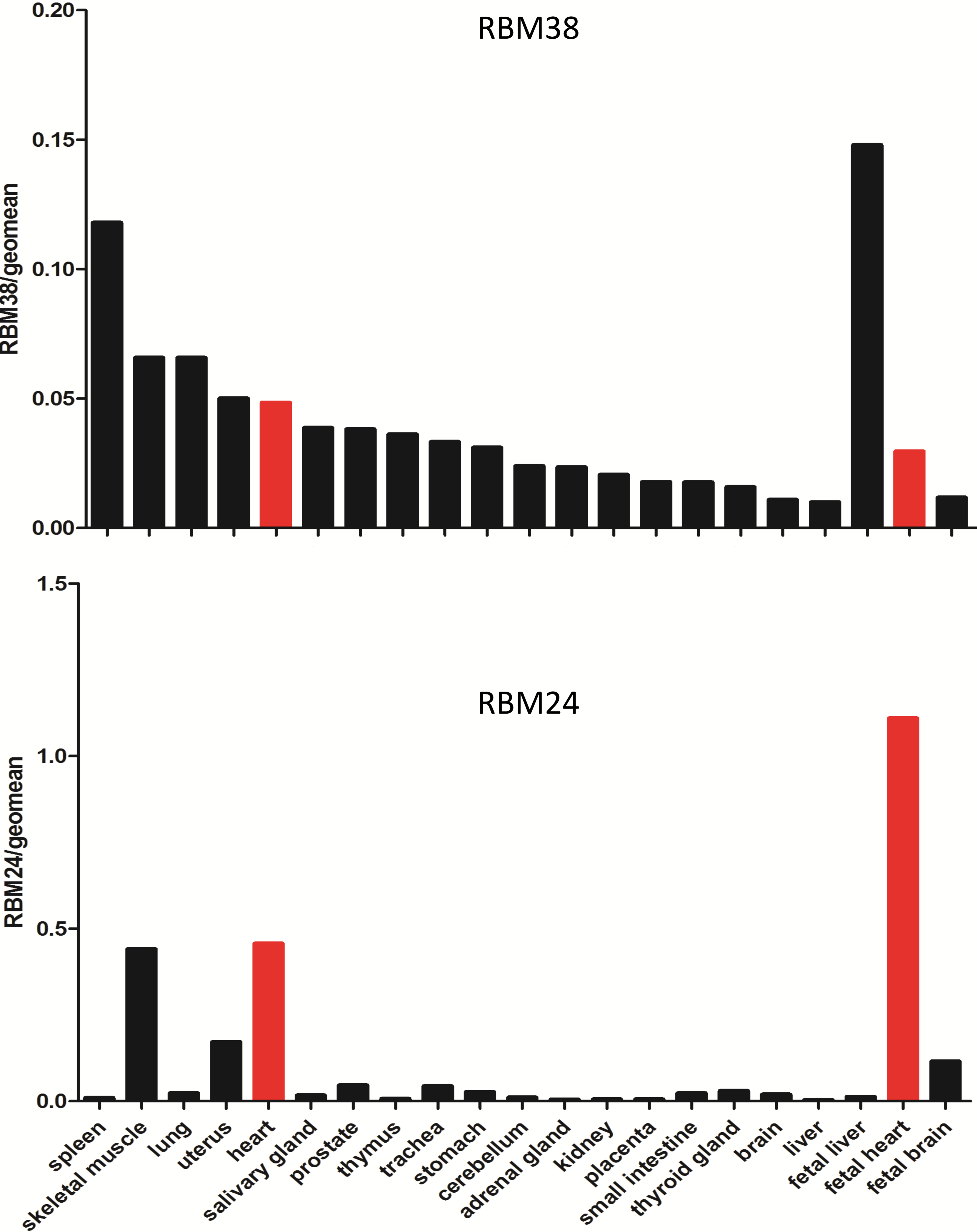

Supplement: S1 Fig — A. qPCR of RBM24 and RBM38 in human RNA tissue panel. Values are corrected for the geometric mean of the following reference genes: GAPDH, HPRT, and B2M. (PDF) [file pone.0184093.s001.pdf]

## S2 Figure

A.

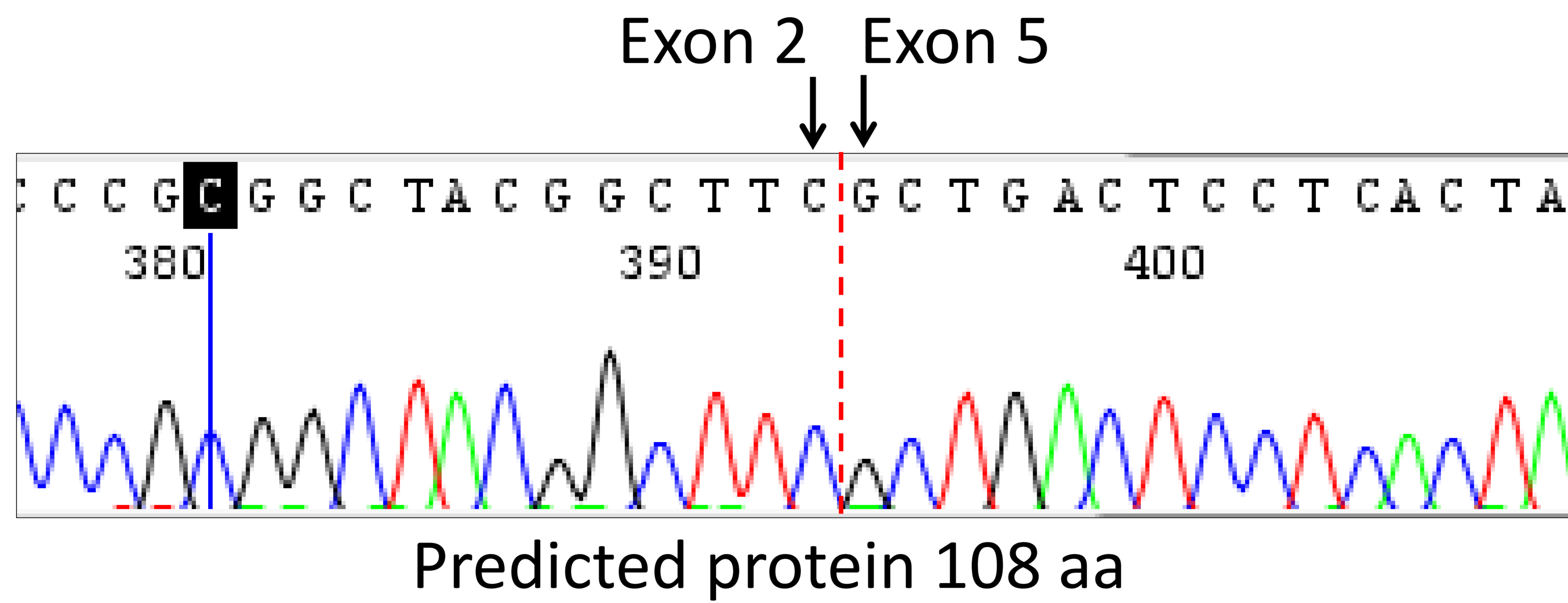

B.

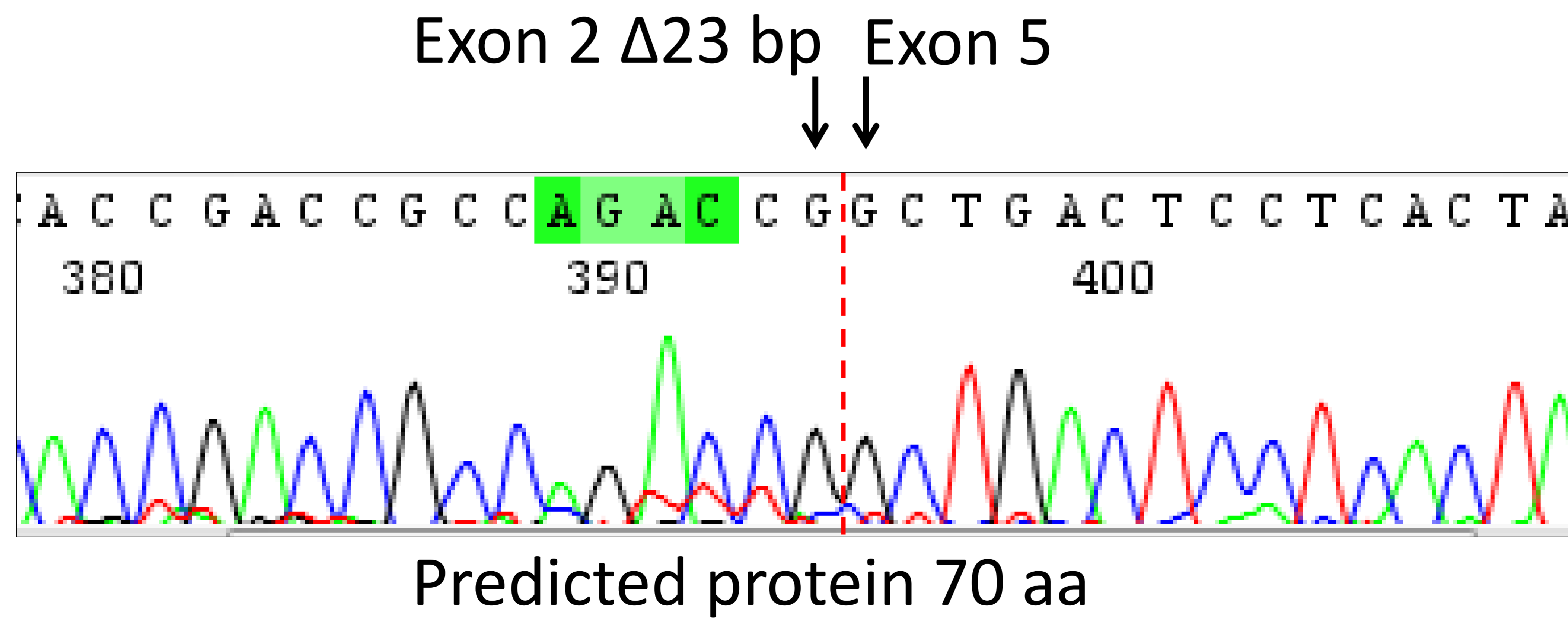

Supplement: S2 Fig — PCR product of Rbm38 -/- hearts in Fig 2A was cloned into pGEM T-easy and 8 clones were Sanger sequenced. A. 7 out of 8 clones showed a splice junction from exon 2 to exon 5 resulting in a transcript that is predicted to produce a protein of 108 amino acids. B. 1 of the 8 clones contained a splice junction of an alternative splice site in exon 2 (23 bp before the end of exon 2) to exon 5, resulting in a transcript that is predicted to produce a protein of 70 amino acids. Dotted red line indicates the splice junction. (PDF) [file pone.0184093.s002.pdf]

S3 Figure

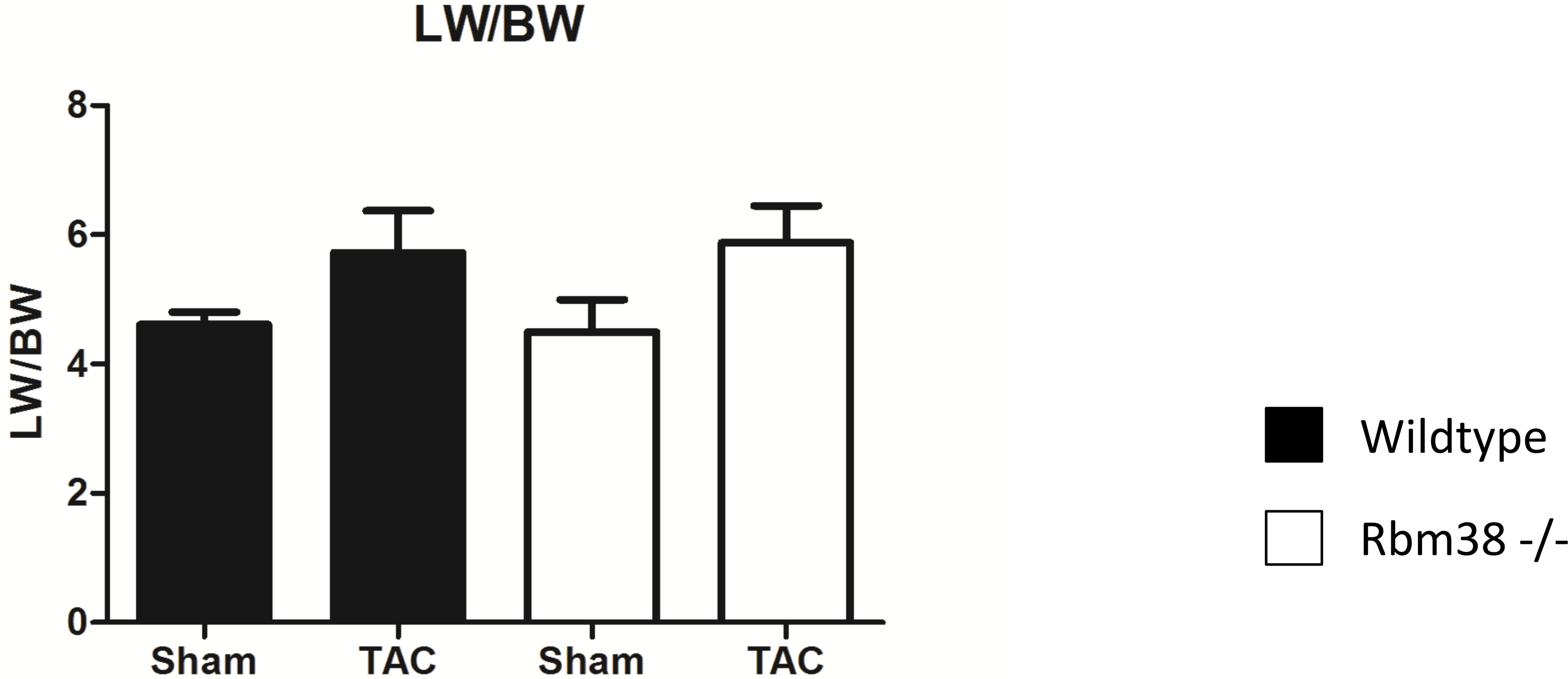

Supplement: S3 Fig — Lung weight/Body weight ratio in sham-operated and TAC-operated wildtype and Rbm38 -/- mice 7 weeks after surgery. (PDF) [file pone.0184093.s003.pdf]

S4 Figure

A.

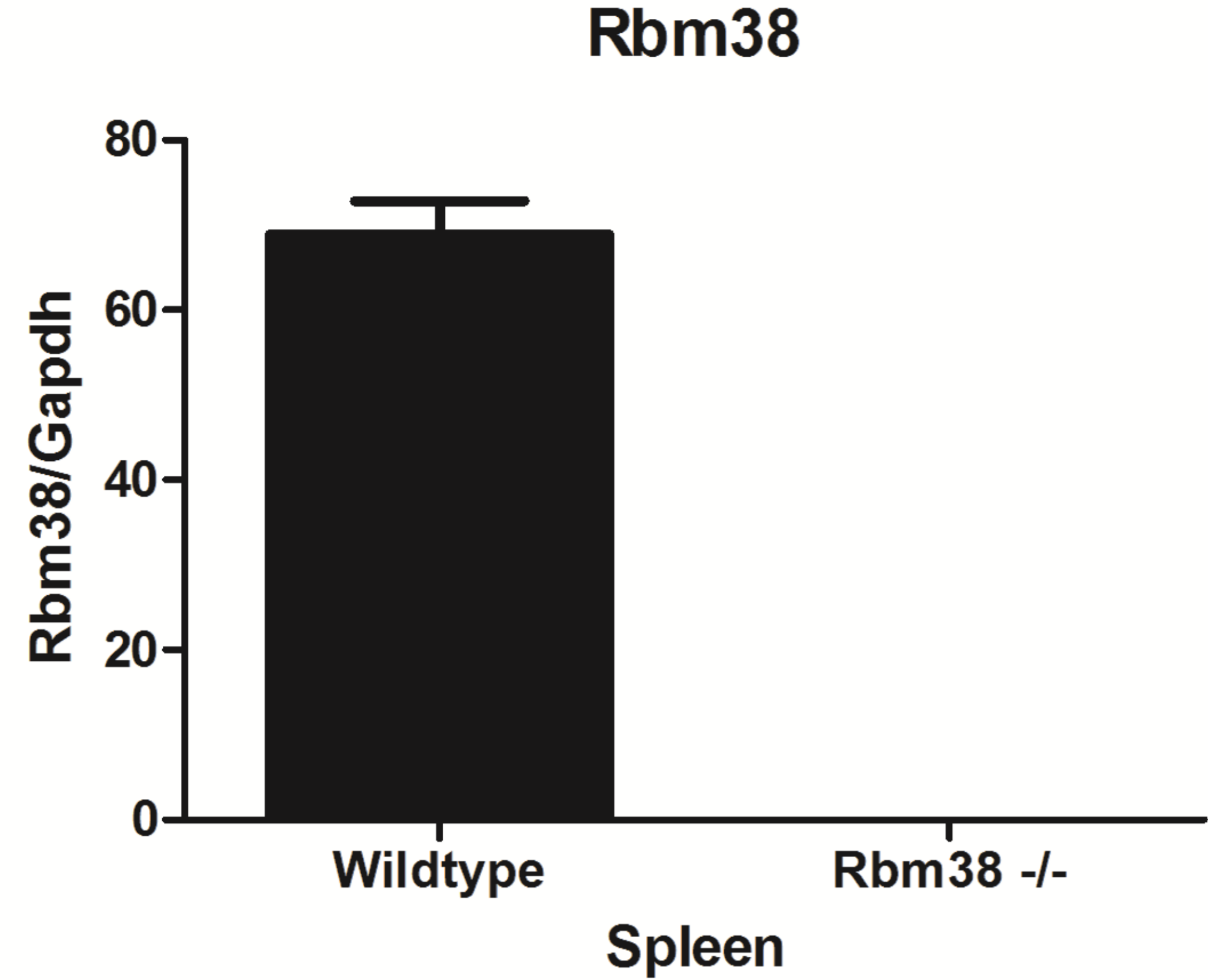

B.

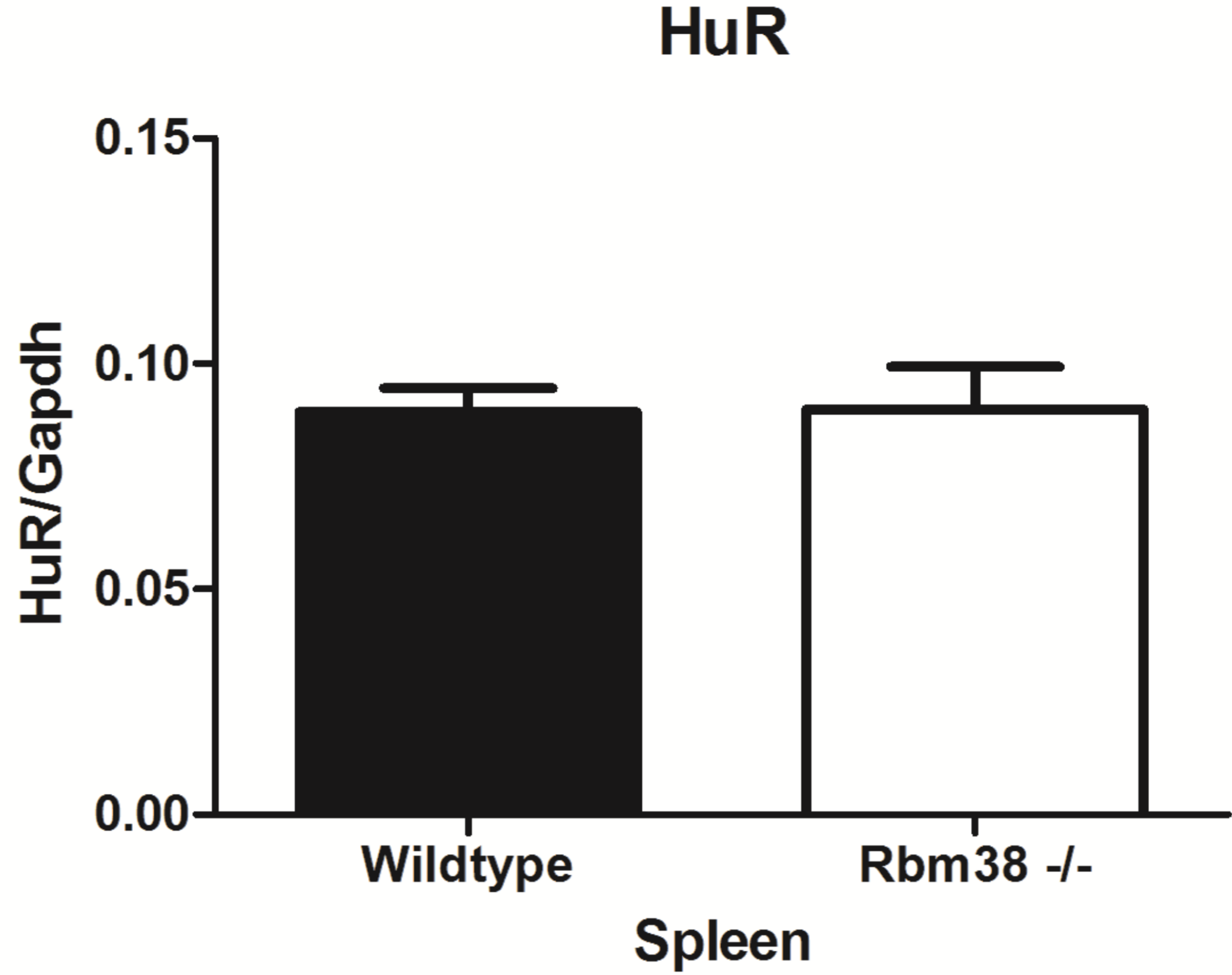

C.

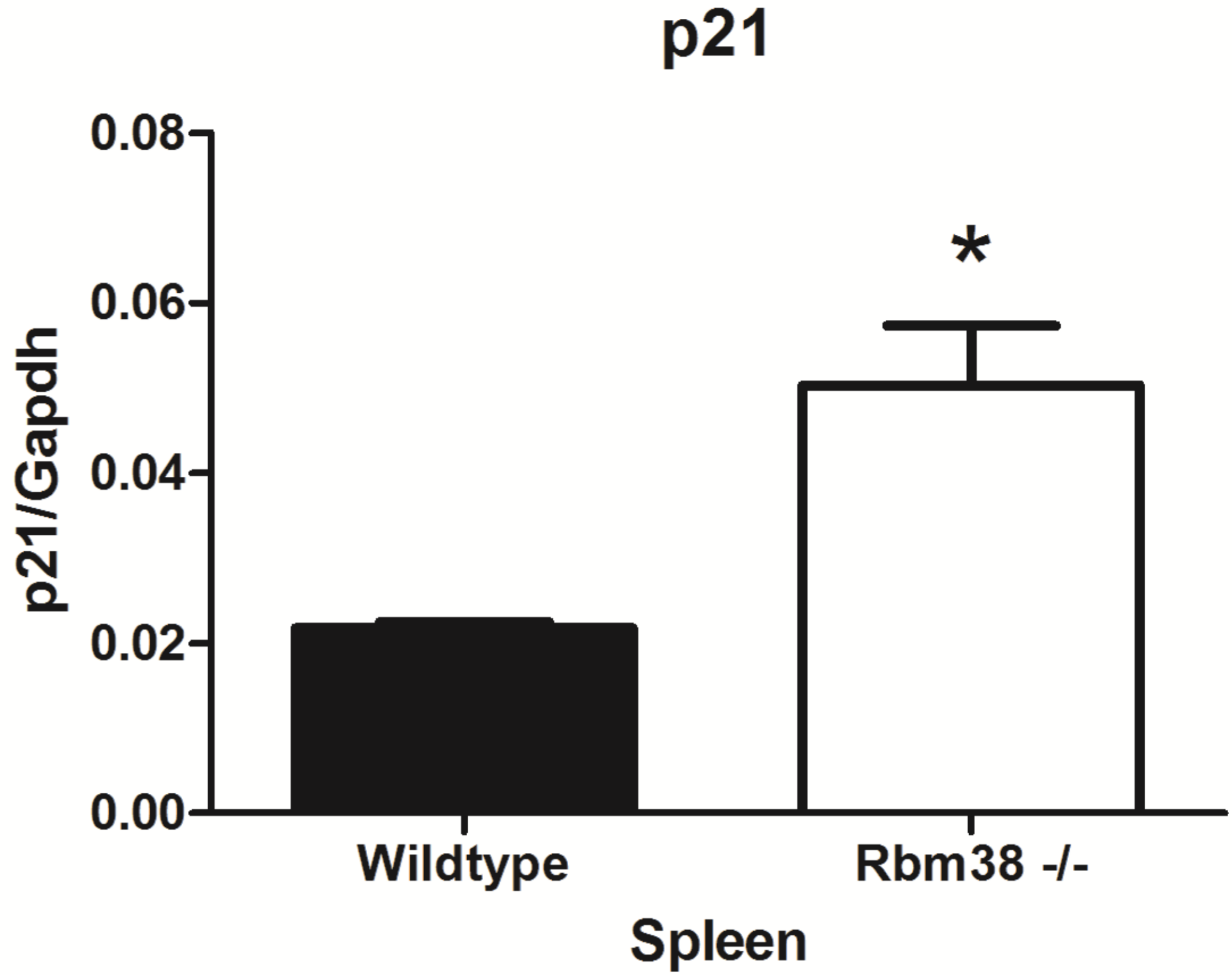

D.

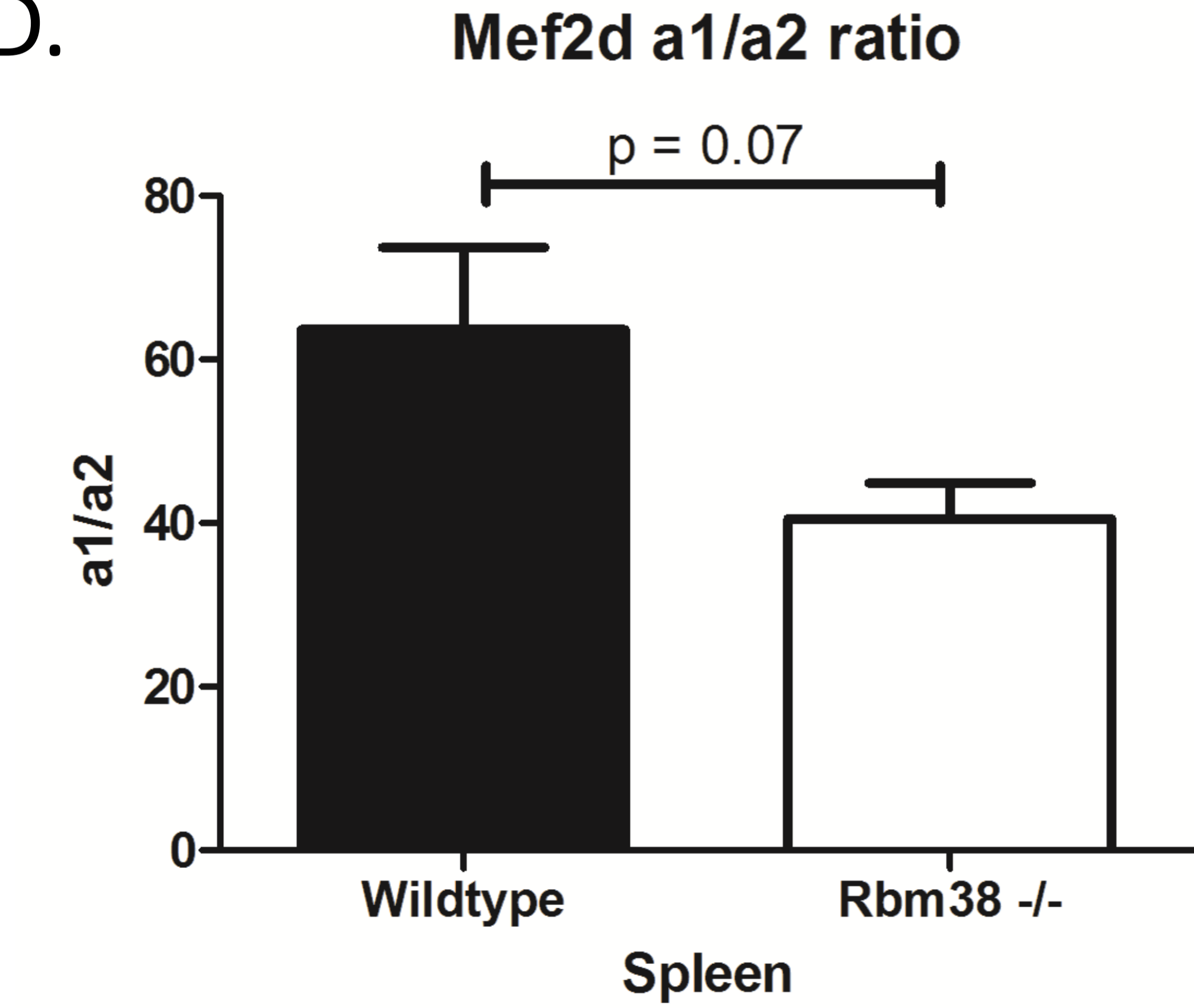

E.

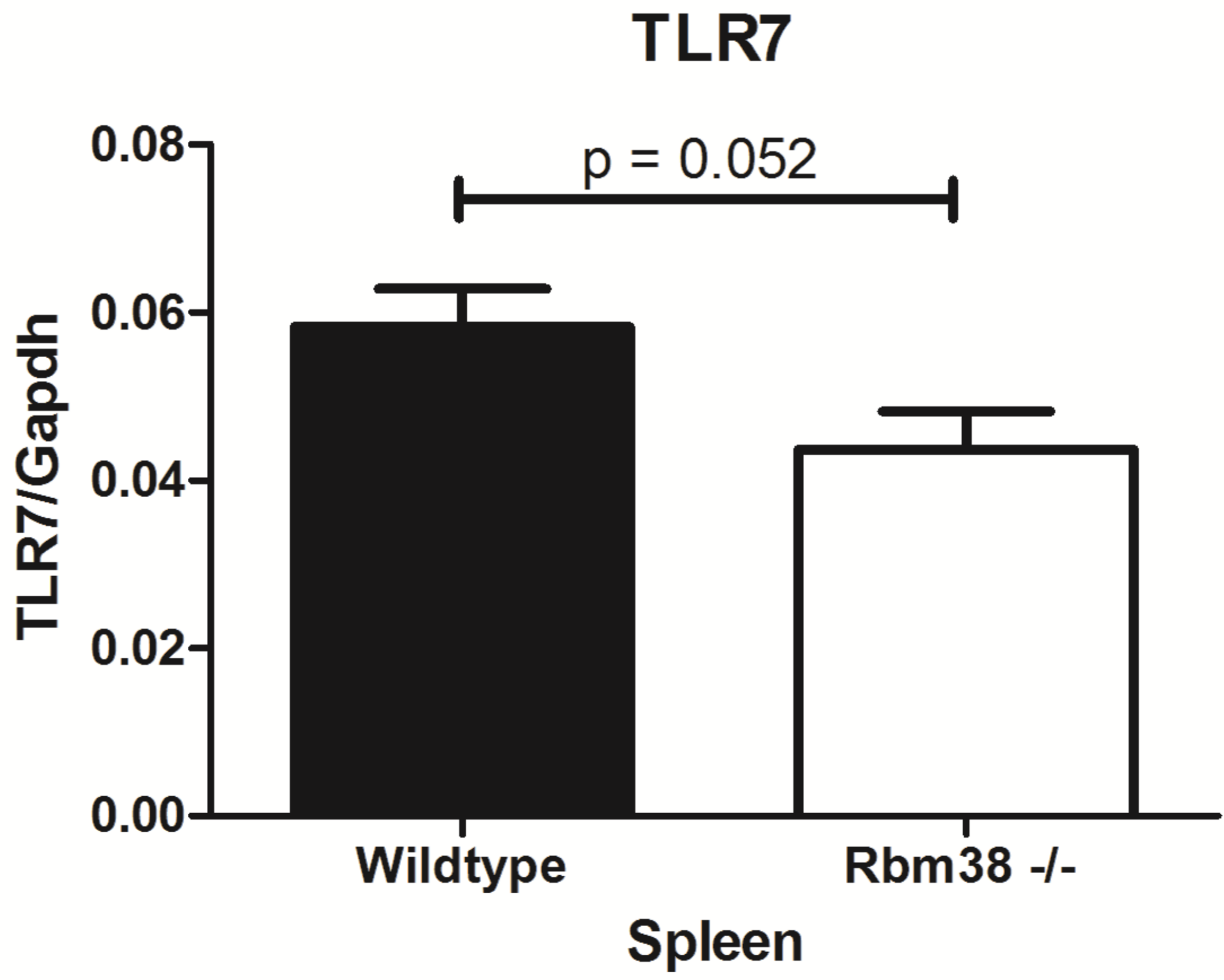

F.

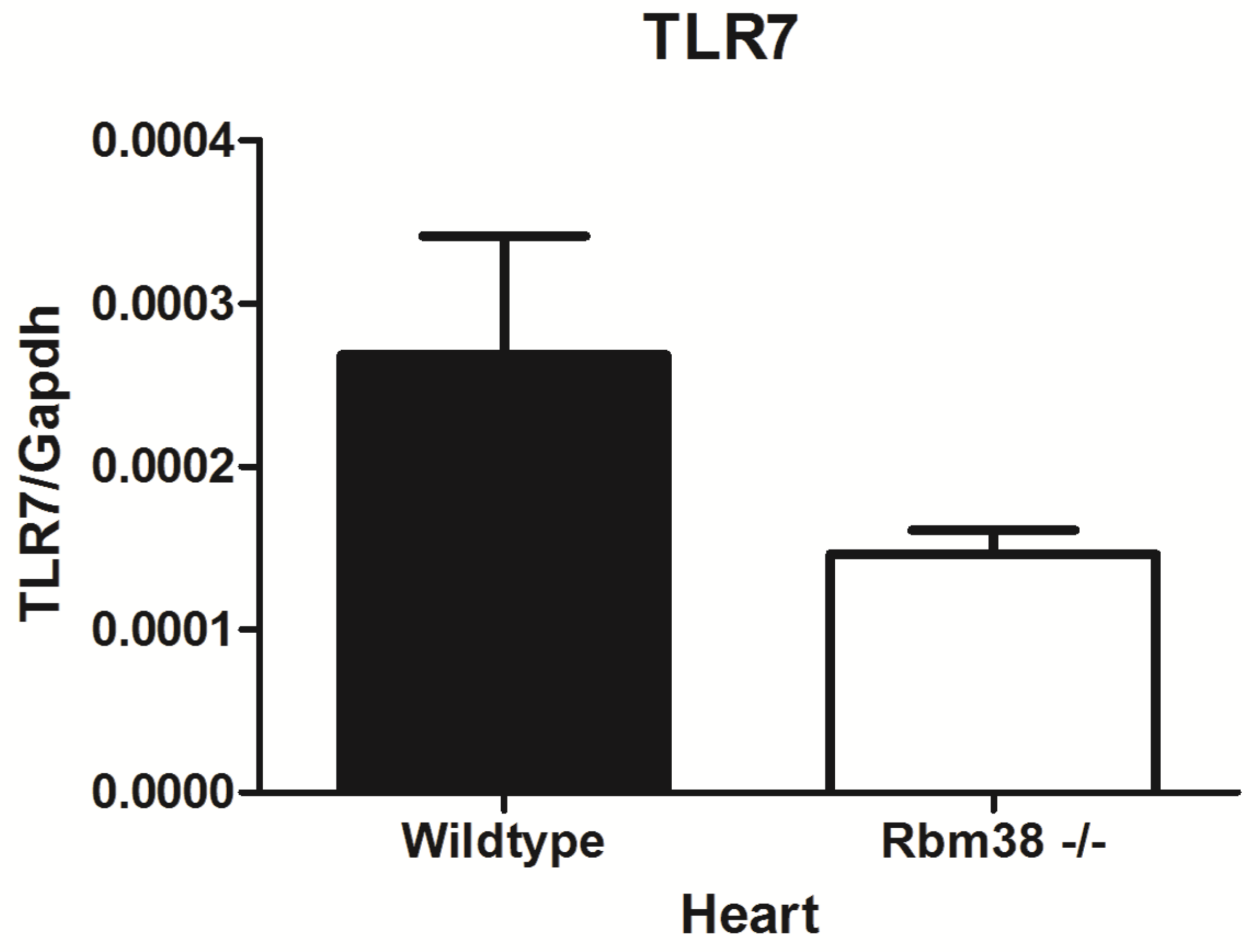

Supplement: S4 Fig — A. qPCR analysis of Rbm38 in wildtype and Rbm38 -/- spleens. B. qPCR analysis of HuR in wildtype and Rbm38 -/- spleens. C. qPCR analysis of p21 in wildtype and Rbm38 -/- spleens. D. qPCR analysis of Mef2d exon α1/ α2 inclusion. E. qPCR analysis of TLR7 in wildtype and Rbm38 -/- spleens. F. qPCR analysis of TLR7 in wildtype and Rbm38 -/- hearts. Wildtype spleens n = 5, Rbm38 -/- spleens n = 5. Wildtype hearts n = 5, Rbm38 -/- hearts n = 3. (PDF) [file pone.0184093.s004.pdf]

S5 Figure

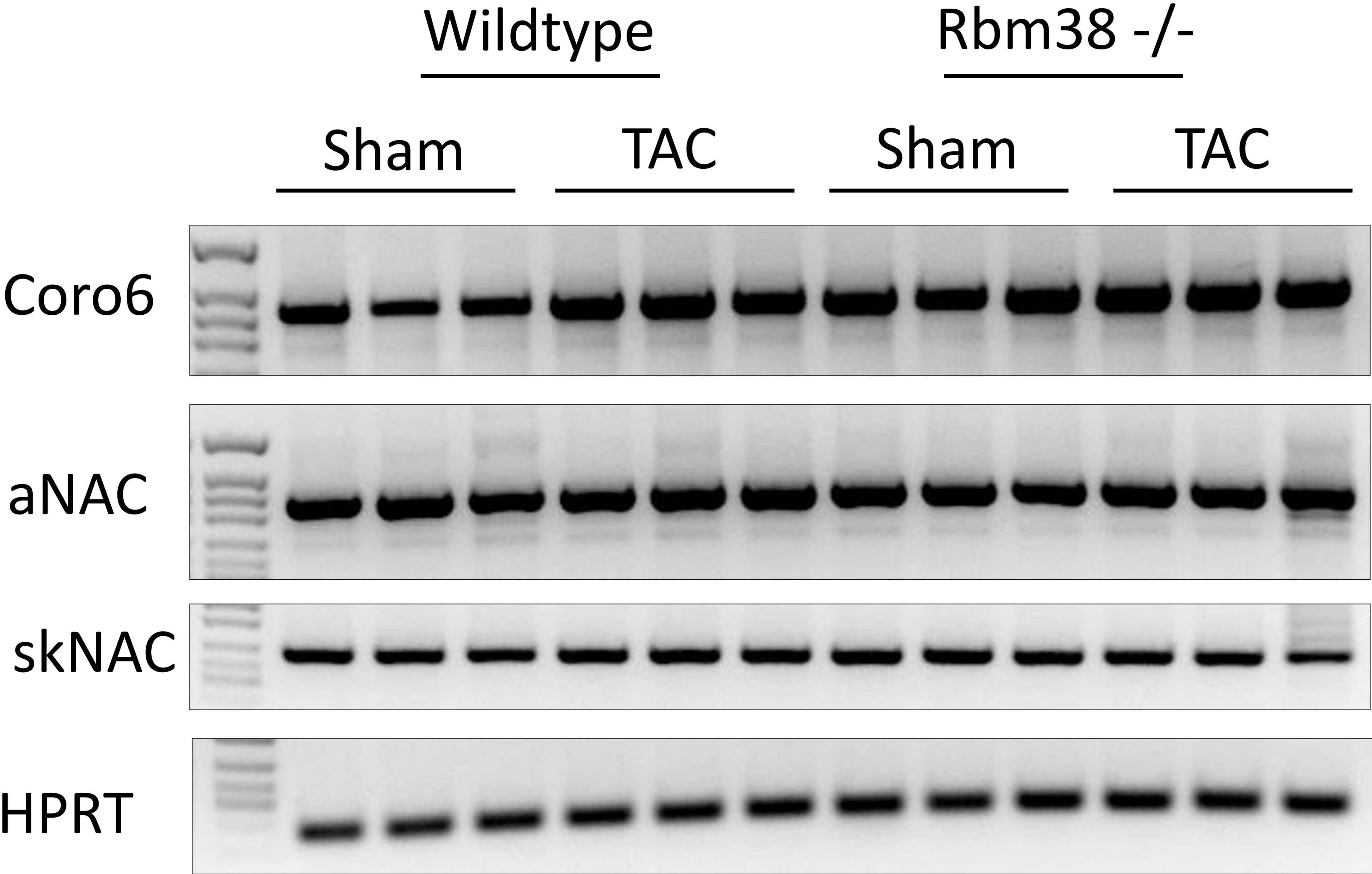

Supplement: S5 Fig — RT-PCR of Rbm24 splicing targets Coro6, aNAC, and skNAC. HPRT was used as a loading control. (PDF) [file pone.0184093.s005.pdf]

S6 Figure

A.

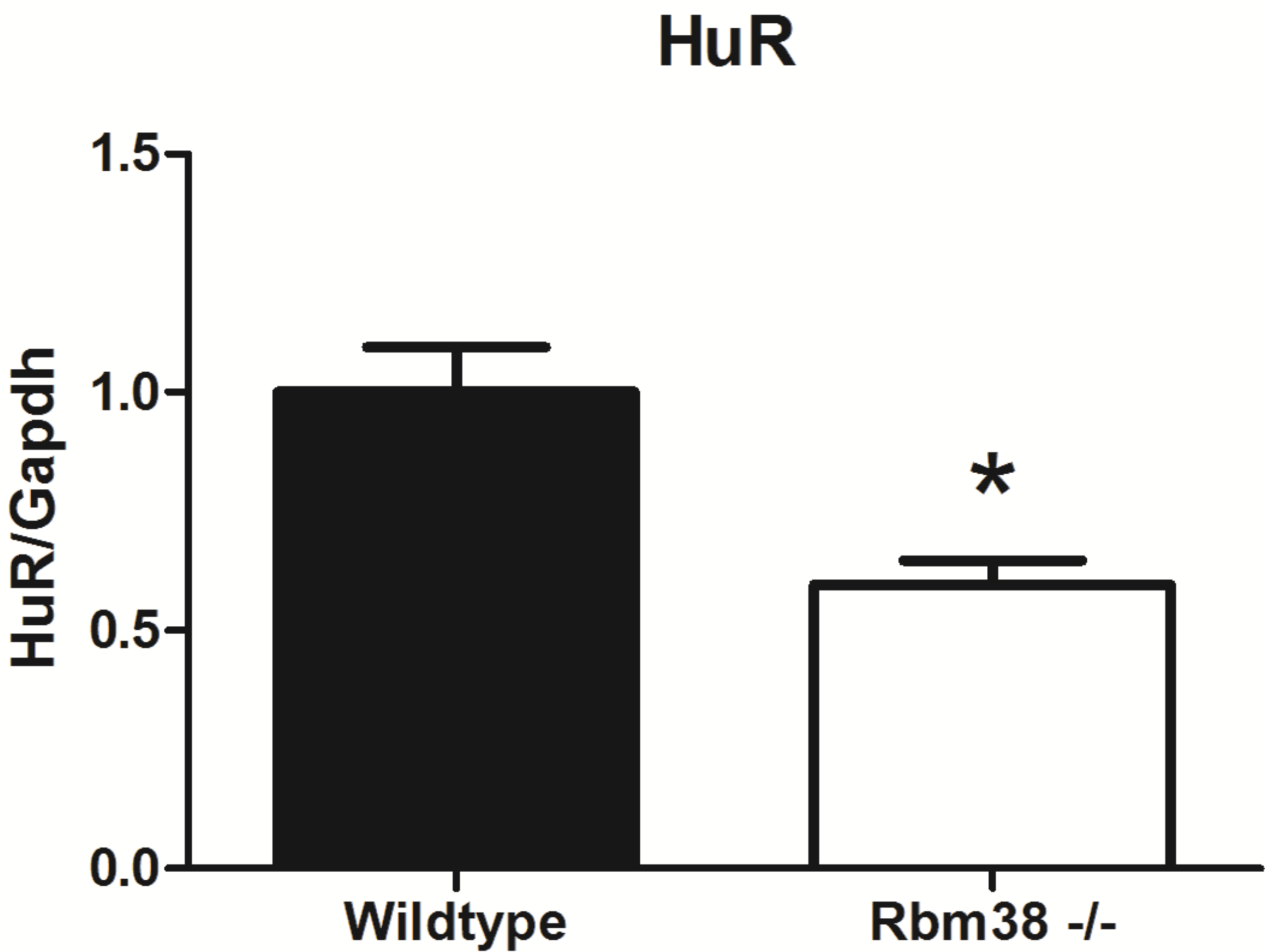

B.

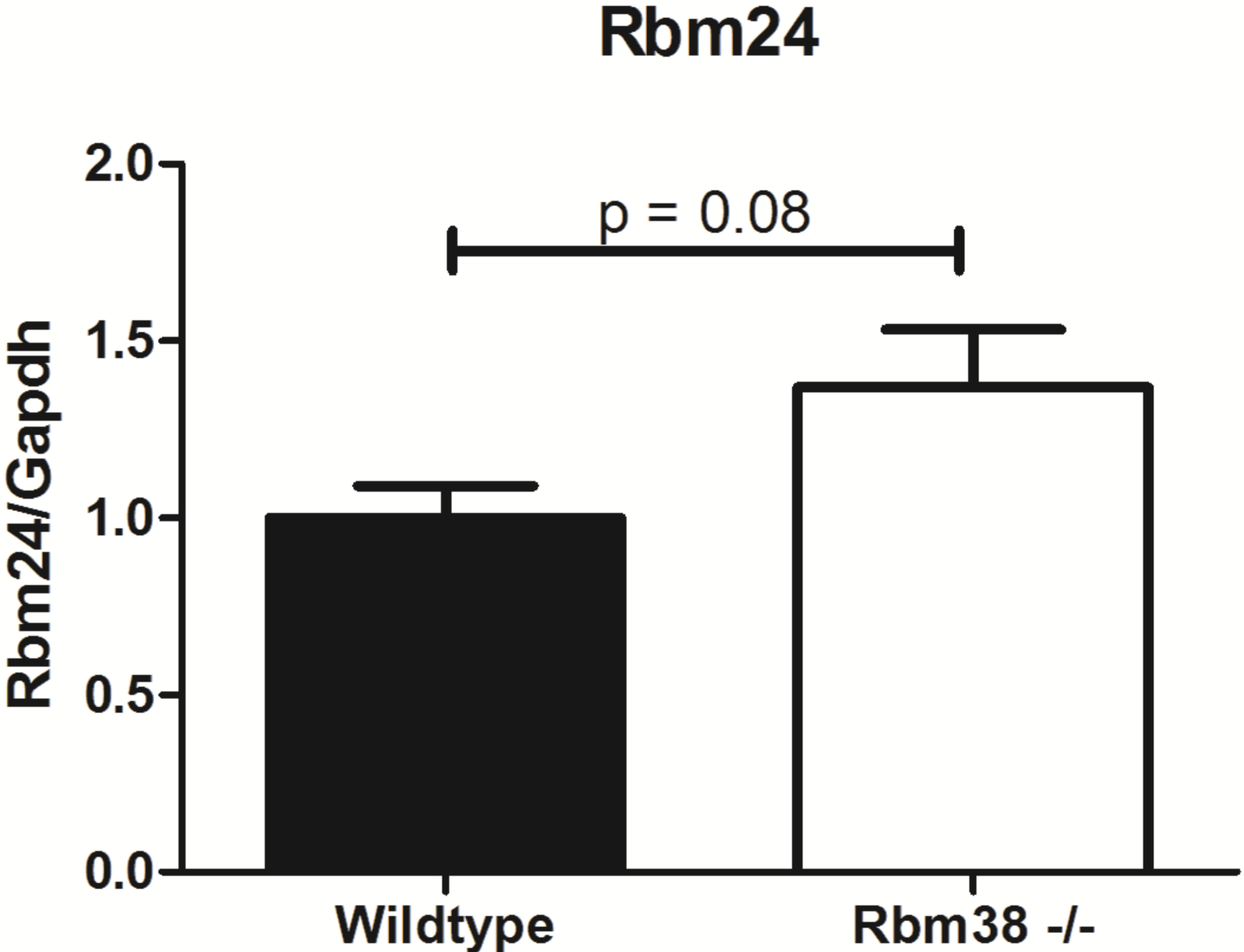

Supplement: S6 Fig — A. Quantification of Western blot in Fig 4H. B. Quantification of Western blot in Fig 5B. (PDF) [file pone.0184093.s006.pdf]
